# From anticipation to memory: a systematic review of tourist emotional experience

**DOI:** 10.3389/fpsyg.2026.1907118

**Published:** 2026-08-07

**Authors:** Jiarong Zeng, Thinaranjeney Thirumoorthi, Mozard Mohtar

**Affiliations:** 1 School of Business, Liming Vocational University, Quanzhou, China; 2 Faculty of Business and Economics, University of Malaya, Kuala Lumpur, Malaysia

**Keywords:** ADO framework, person-environment interaction, research agenda, systematic review, tourism environments, tourist emotional experience

## Abstract

Tourist emotional experience has emerged as a central concept in understanding how individuals psychologically engage with tourism environments. It is reflects a broader shift from functional attributes to experiential and affective dimensions of travel. However, existing research tends to focus on isolated emotions or single stages of the tourist journey. It therefore lacks a dynamic perspective. This study addresses this gap through a systematic review of 211 peer-reviewed articles (2000–2025), applying the antecedents-decisions-outcomes (ADO) framework to synthesize tourist emotional experience across pre-, during-, and post-visit phases. This review adopts an environmental psychology perspective to conceptualize tourist emotional experiences as emerging from dynamic person-environment interactions, where tourism environments spanning physical, sensory, symbolic, social, and digital dimensions function as multidimensional stimuli. The findings show a clear temporal pattern in tourists’ emotional development, with anticipation before the visit shaped by destination environmental cues and informational influences, on-site engagement by personal psychological states, and post-visit loyalty and memory by social interactions. This study also highlights the growing influence of immersive technologies (e.g., VR/AR) and crisis-related factors on emotional trajectories. By integrating a journey-based perspective, this study advances understanding of how emotional experiences unfold across travel stages and offers implications for designing effective emotional touchpoints.

## 1 Introduction

From an environmental psychology perspective, tourist experiences are shaped by continuous interactions between individuals and their environments (Santos et al., 2022; Gifford, 2014). These environments nowadays extend beyond physical settings to include sensory, symbolic, and increasingly digital environments, all of which function as environmental stimuli that shape tourist emotional experiences and influence their subsequent behavior (Wu and Zhao, 2023; Dutta and Dixit, 2025). Building on this perspective, the ability of environments to evoke emotional responses becomes particularly significant in the context of intensifying global competition among tourism destinations. Contemporary destination research faces clear limitations in relying solely on traditional forms of differentiation such as physical attractions, infrastructure, or service attributes (Wu and Lai, 2023). The increasing standardization of tourism offerings has resulted in homogenized and similar destination experiences, accelerating the commodification of tourism products. Under these conditions, differentiation based on functional attributes alone is no longer sufficient (Kim et al., 2024). Instead, destinations are increasingly required to adopt experiential forms of distinction, in which environments are strategically designed to evoke specific emotional responses.

In this context, emotional experiences have emerged as an important source of competitive advantage (Jiang and Garrod, 2025). Given the growing emphasis on emotions in destination studies, destination management organizations (DMOs) have increasingly use storytelling as a symbolic environmental stimulus to create emotionally engaging narratives that resonate with travelers seeking authentic experiences (Ma et al., 2024). Some destinations further enhance emotional engagement through visual and sensory stimuli designed to elicit specific affective responses, such as tourism videos (Liu et al., 2025) and multi-sensory auditory cues (Yin et al., 2025). Social media has amplified these effects by functioning as a digital environment that enables user-generated storytelling, allowing emotional meanings to extend beyond official promotional content and strengthening tourist engagement (Sykora et al., 2022). Technological advances have further intensified these dynamics. The integration of virtual and augmented reality has enabled destinations to create immersive digital environments that support more personalized and emotionally engaging experiences (Li et al., 2025). Overall, these developments highlight the growing importance of environment-based emotional experience design in destination differentiation.

Despite the growing attention to tourist emotional experiences in destination research, a comprehensive understanding of how emotional experiences unfold and influence tourist behavior remains limited due to the complex and dynamic nature of emotions. Specifically, emotional experiences continuously evolve throughout the tourist journey (Godovykh and Tasci, 2021), reflecting ongoing interactions between individuals and changing environmental stimuli. Hence, they are highly individualized, shaped by tourists’ personal characteristics, cultural backgrounds, and situational contexts (Hosany et al., 2020; Santos et al., 2022). Such complexity makes emotional experiences difficult to predict, measure, and manage through one-size-fits-all strategies. Given this phenomenon, tourist emotional experiences can be better understood through a process-based framework that examines how diverse tourism environmental antecedents trigger emotions, shape decision-making, and drive behavioral outcomes throughout the entire journey. Despite this need, current literature lacks such a holistic perspective. To date, no systematic review has rigorously synthesized tourist emotional experiences through a structured organizing framework like ADO (antecedents, decisions, and outcomes) that maps these interconnected elements.

Current literature exhibits two primary limitations. First, existing reviews lean heavily toward a single-emotion lens that concentrate on disappointment, awe, or nostalgia (Michalkó et al., 2015; Wang et al., 2021; Thanh and Dung, 2025). While these studies offer significant insights into specific emotions’ generation processes, antecedents, or consequences, they fail to capture the dynamic and multifaceted tourist’s emotional experiences. From a theoretical perspective, tourists’ experiences are complex, characterized by multiple co-occurring emotional states throughout travel journey (Hosany and Gilbert, 2010; Prayag et al., 2013). By isolating individual emotions, current research fails to capture how emotional experiences unfold across different touchpoints, thereby limiting the understanding of the full emotional experiences of the tourists’ journey. Second, and more fundamentally, existing research on tourists’ emotional experiences tends to examine antecedents and behavioral outcomes separately, rather than as parts of an integrated process. Previous studies examined either on factors influencing emotional experiences (Aro et al., 2018; Elbaz et al., 2023) or the consequences of emotions on tourist behaviors (Kim et al., 2022; Rezaei et al., 2023; Lin et al., 2024) but none within a unified framework. This fragmented approach obscures the critical mediating role of decision-making processes that bridge antecedents and outcomes. Past research has rarely examined how various tourism environmental antecedents jointly trigger emotional experiences and how these emotions shape tourists’ decisions across journey stages, including pre-visit planning, during-visit adaptations, and post-visit reflections, ultimately leading to behavioral outcomes. Given the complexity of tourist emotional experiences, scholars have called for more comprehensive research into how these experiences unfold across the tourist journey (Zhao and Agyeiwaah, 2023; Volo, 2021). This requires a holistic understanding of how environmental antecedents, internal decision processes, and behavioral outcomes interconnect within destination contexts. Existing reviews have not employed a structured framework that positions emotional experiences as the central focus of analysis. Without such systematic mapping of the complete emotional journey there will be lack of theoretical clarity on how emotional experiences could unfold across the tourist journey. This gap also limits the development of practical insights into how emotional touchpoints can be effectively designed to foster desired destination management outcomes.

To address these gaps and respond to recent calls for a more nuanced and systematic understanding of emotional experiences as a core research domain in destination studies (e.g., Hosany et al., 2022; Volo, 2021; Ma et al., 2024), this review adopts the ADO framework, which conceptualizes tourist emotional experiences through their antecedents, decision processes, and outcomes (Paul et al., 2023). The ADO framework is particularly suitable as it reflects the causal structure of tourist behavior and enables a systematic integration of fragmented findings across the tourist journey (Lim et al., 2021). It is also conceptually aligned with the Stimulus–Organism–Response logic in environmental psychology, as both frameworks capture the progression from external conditions to internal processes and behavioral outcomes (Mehrabian and Russell, 1974; Gifford, 2014). Building on this alignment, this review synthesizes theoretical and empirical research on tourist emotional experiences in destination contexts published between 2000 and 2025. By organizing the literature through the ADO framework, the study clarifies how environmental stimuli shape tourist emotional experiences, how these experiences influence decision-making, and how they translate into behavioral outcomes. Accordingly, this study addresses the following research questions:

*RQ1*: What environmental antecedents shape tourist emotional experiences across different stages of the travel journey?

*RQ2*: How do emotional experiences shape tourist decisions across different stages of the travel journey?

*RQ3*: What outcomes result from tourist emotional experiences?

*RQ4*: What are the future directions for advancing the understanding of tourist emotional experiences?

This systematic review contributes to both tourist emotional experience research and environmental psychology in several ways. First, it shifts the analytical focus from isolated emotions to holistic emotional experiences. In contrast to reviews focused on isolated emotions (Wang et al., 2021; Thanh and Dung, 2025), this study approaches the tourist emotional experience as a broader research domain, tracing its evolution from pre-visit anticipation and on-site engagement through to post-visit reflection. To ensure this focus, the review includes studies in which emotional experience is the central theoretical or empirical concern and which provide insights into how emotions shape tourist decisions and outcomes across different stages of the journey. This perspective addresses the limitations of single-emotion studies which offer limited guidance for destination management. For instance, while excitement may encourage adventure activity participation, concurrent anxiety about safety ultimately determines the actual behavior. By viewing emotional experiences as integrated processes, this review reveals how they evolve through different journey stages, enabling DMOs to manage emotional touchpoints strategically rather than optimizing isolated moments. Second, this review contributes to environmental psychology by expanding its focus beyond traditional physical settings to include hybrid environments that combine sensory, symbolic, and digital elements. It shows that digital environments, such as social media, virtual reality, and online visual content, act as important digital environmental stimuli that shape tourists’ emotional experiences beyond on site contexts. By incorporating emerging forms of tourism environments, this review extends environmental psychology perspectives by broadening the range of stimuli considered in explaining tourist emotional responses and subsequent behaviors. Third, this review first adopts the ADO framework to conceptualize tourist emotional experiences in terms of antecedents, decisions, and outcomes, where antecedents refer to environmental stimuli that trigger emotional experiences, decisions capture how emotions shape tourist choices across journey stages, and outcomes refer to resulting behaviors. This framework provides DMOs with actionable guidelines for designing and managing emotional touchpoints at each stage of the tourist journey. From an environmental psychology perspective, this structured synthesis offers a process-based understanding of how different environmental factors interact to shape tourist emotional experiences across the travel journey.

The remainder of this paper is structured as follows. First, the review on methodology and procedures are presented and followed by main findings based on ADO framework which explain “what do we know” about tourist emotional experience. Next, suggestions for future research are proposed based on the ADO-based analysis by shedding light on “where the field should be heading” (Lim et al., 2021). Finally, the paper concludes by summarizing the key insights of the review and discuss the theoretical and practical implications for research and destination management practice.

## 2 Methodology

### 2.1 Review method

This study applied a systematic literature review (SLR), as it recognized for its capacity to rigorously synthesize existing knowledge while maintaining procedural transparency and replicability (Snyder, 2019). The decision to employ an SLR was driven by two strategic considerations. First, the systematic reviews enable researchers to move beyond narrative summaries by applying explicit, reproducible protocols for study identification and evaluation (Petticrew and Roberts, 2006). Second, this approach can map the knowledge and uncover critical research gaps for future research (e.g., Rosalina et al., 2021; Zhang and Ramayah, 2024).

Designing a systematic review requires a clear choice of synthesis strategy due to methodological issues such as potential bias in study selection, challenges in comprehensive literature search, and heterogeneity in study designs and evidence types (Lame, 2019). Paul et al. (2021) identified five types of reviews namely domain, theory, methodology, meta analytical and meta-systematic. Domain reviews, which aim to advance substantive knowledge by synthesizing and organizing empirical findings within a specific research field (e.g., a topic such as tourist emotional experiences) (e.g., Lyu et al., 2023). Theory reviews, which focus on examining, refining, and extending existing conceptual frameworks to promote theoretical development (e.g., Sop, 2020). Methodology reviews, which assess research designs, data collection techniques, and analytical approaches in order to identify methodological patterns (e.g., John and Sivakumari, 2024). Meta-analytical reviews, in which scholars apply statistical techniques to systematically analyze empirical evidence and estimate overall relationships among variables (e.g., Luo et al., 2025). And finally, meta-systematic reviews, which review prior review studies and critically evaluate the scope, quality, and conclusions of existing reviews, thereby providing a meta-level assessment of accumulated knowledge (e.g., Bond et al., 2024).

**Figure 1:**
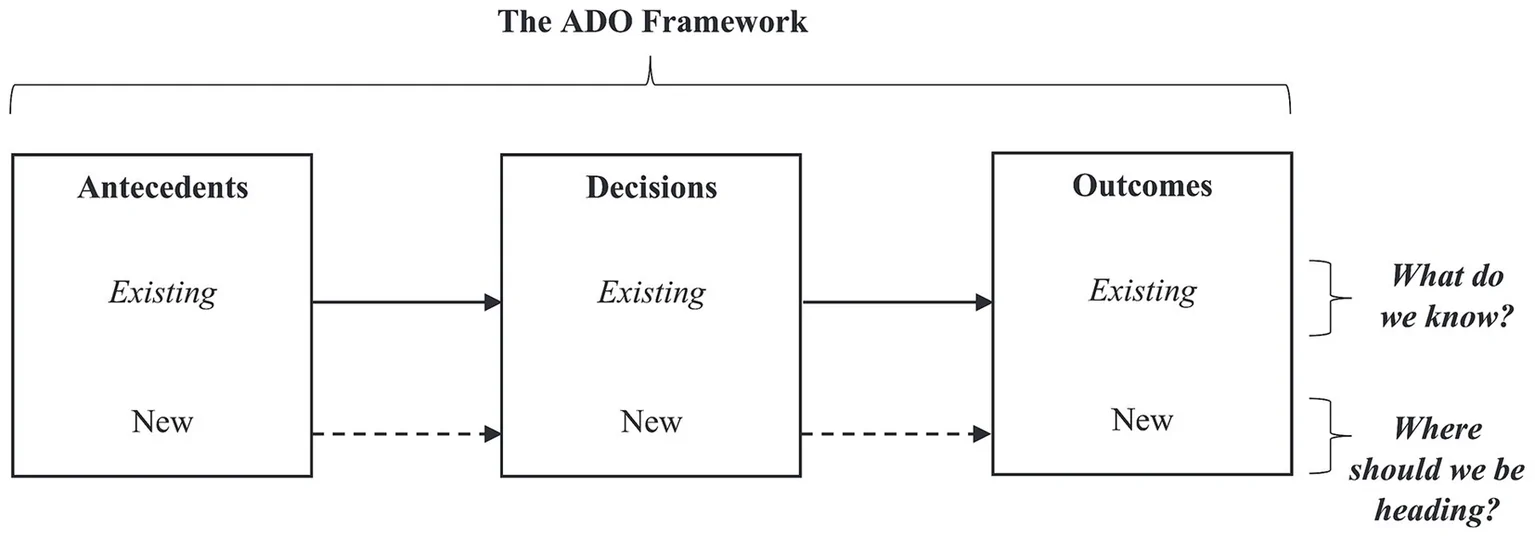
The ADO framework (adapted from Paul and Benito, 2018; Lim et al., 2021).

Specifically, this study employs a domain-based systematic literature review to address the research objectives, as research on tourist emotional experiences in destination contexts remains fragmented and lacks a coherent and structured overview. Within this study, tourist emotional experience is conceptualized as a research domain referring to tourists’ affective responses and emotional states that arise from their interactions with physical, social, and digital environments across the travel journey. Following Lim et al. (2021) guidelines, the review was conducted in two stages. First, the PRISMA protocol was utilized in the first phase to screen and select relevant studiesto ensure procedural rigor and reproducibility (Moher et al., 2009). In the second phase, the selected articles were synthesized using the ADO framework to map the temporal progression of tourist emotional experiences from pre-visit antecedents and on-site decision-making to post-visit outcomes. The synthesis of studies across these two stages reconstructs the evolution of emotional experiences throughout the tourism journey providing a longitudinal understanding of emotional dynamics. Figure 1 presents the ADO framework used in this review.

### 2.2 The PRISMA protocol

This study followed the PRISMA protocol, including the four stages of identification, screening, eligibility, and inclusion, to systematically select relevant literature. PRISMA was adopted to ensure transparency, reproducibility, and rigor in the review process, and to minimize potential selection bias (Shamseer et al., 2015).

#### 2.2.1 Identification

During identification stage, Web of Science (WoS) and Scopus were used as two sources. The former served as the primary database due to its rigorous journal indexing and broad coverage of peer-reviewed academic publications (Zhang et al., 2022) while the latter complemented retrieval to reduce omissions. The search employed a comprehensive string using (“emotion” OR “emotional experience”) AND (“destination” OR “tourism”) as primary keywords, combined with expanded terms such as “emotional response,” “emotional engagement,” “emotional attachment,” and “emotional bond” to capture the diverse expressions of tourist emotional experiences. The review covered publications from 2000 January 1st to December 31st, 2025 to capture both foundational and emerging studies. Only peer-reviewed English-language journal articles were retained. The initial search yielded 1,003 records where 307 and 696 were from WoS and Scopus, respectively. The detailed search criteria and corresponding results are summarized in Appendix 1.

#### 2.2.2 Screening

In the screening stage, 160 duplicate records were first removed, leaving 843 articles for further evaluation. The quality of these journals was assessed using the Australian Business Deans Council (ABDC) Tourism Journal Quality List (Appendix 2), which is widely recognized in tourism research as a benchmark for journal ranking (Lim and Rasul, 2022). The ABDC list includes many journals indexed in major citation databases such as the Social Sciences Citation Index (SSCI) and Journal Citation Reports (JCR), thereby ensuring that the selected articles are drawn from reputable and high-quality sources. This step led to the exclusion of 303 articles. Subsequently, relevance to the research topic was examined and this assessment was guided by three criteria: (1) positioning tourists’ emotional experiences as the central research focus rather than as peripheral variables; (2) research questions or objectives directly addressing tourists’ emotional experiences in tourism contexts, and (3) substantive empirical or conceptual analyses examining the antecedents, processes, or consequences of tourist emotional experiences. Based on these criteria, a further 246 articles were excluded. In addition, eight additional articles of systematic reviews (e.g., meta-analysis) were removed to avoid redundancy (Lim and Rasul, 2022).

#### 2.2.3 Eligibility

During the eligibility stage, full-text articles were assessed against predefined inclusion and exclusion criteria to ensure methodological rigor and relevance to the research objectives. Overall, 557 articles were excluded during the screening process, resulting in a final sample of 286 articles that were systematically recorded in a structured Excel database for subsequent analysis (Palocsay et al., 2010). During full-text assessment, studies were further evaluated in terms of methodological rigor (Harrison et al., 2021) and the centrality of their research focus, which led to the removal of 85 articles. Consistent with prior systematic review practices that combine journal rankings with content-based evaluation, a small number of highly cited and relevant studies from reputable journals outside the ABDC categories were retained to ensure comprehensive coverage of the domain.

#### 2.2.4 Inclusion

**Figure 2:**
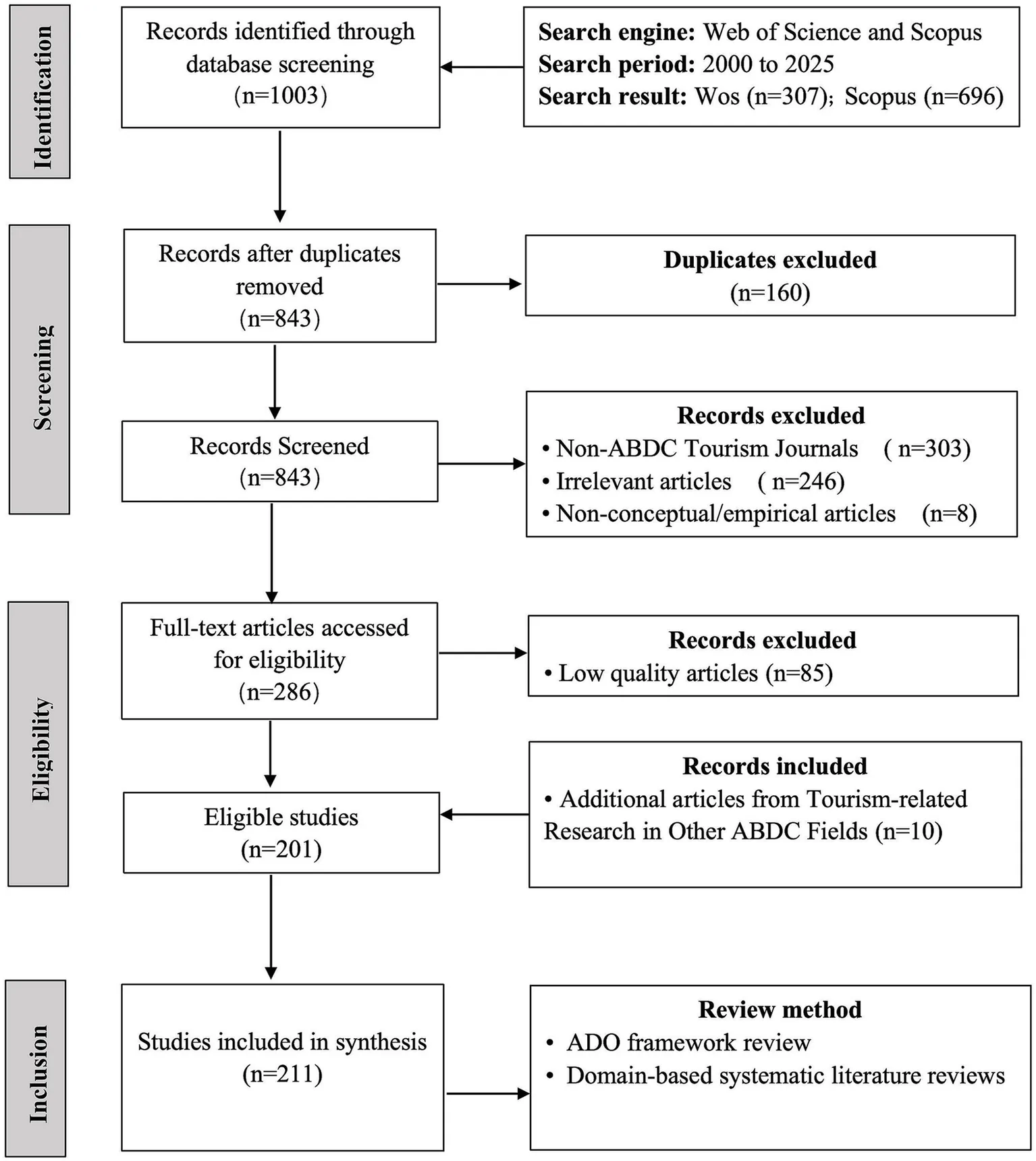
PRISMA flow diagram of the article search and selection process. Articles were collected up to December 31, 2025.

The inclusion stage identified the final set of studies that met all eligibility criteria and were deemed suitable for synthesis and analysis. The final dataset comprised 211 peer-reviewed articles (see Appendix 3), published between 2000 and 2025 across 37 journals. The full selection process is presented in Figure 2.

## 3 What do we know?

**Figure 3:**
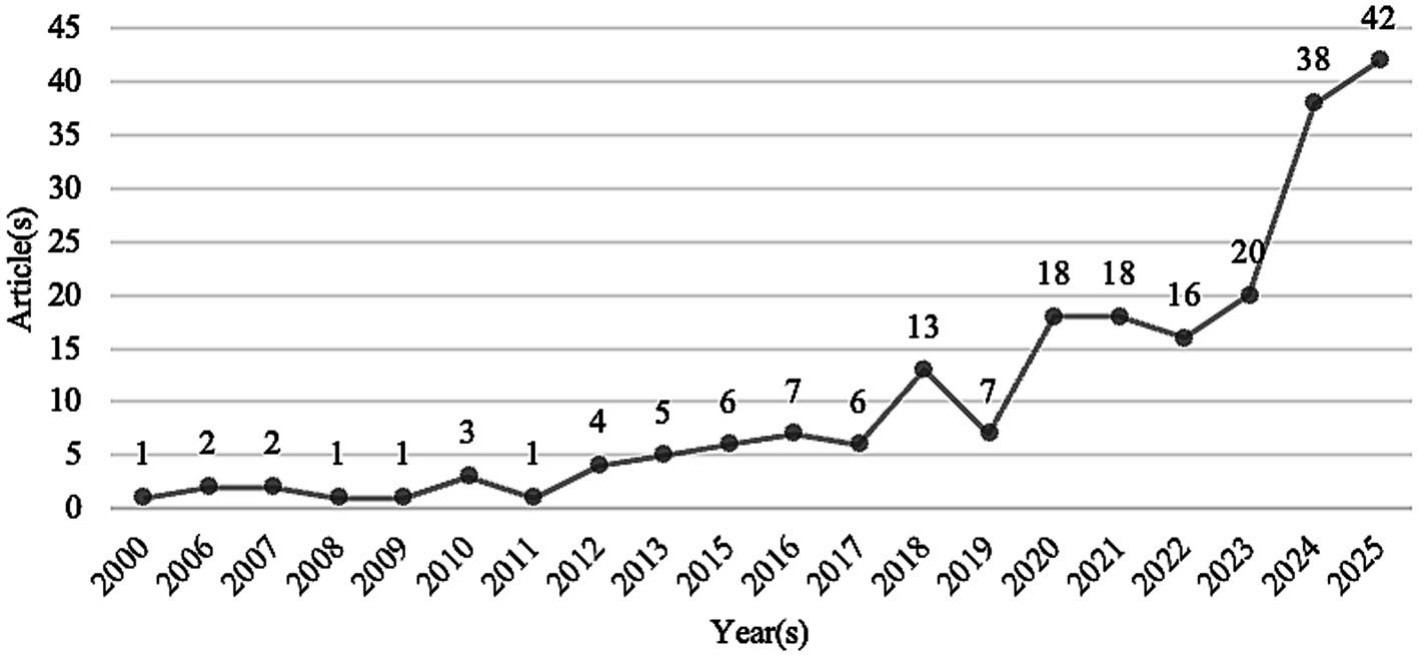
Articles included in the review (*n* = 211). As of December 31, 2025.

As shown in Figure 3, scholarly interest in tourists’ emotional experiences in destination research has increased steadily over the past two decades. Prior to 2010, research in this area was very limited. From 2011 onward, publication output began to grow gradually with a marked acceleration after 2018. Despite minor fluctuations between 2019 and 2022, recent years have seen a sharp increase in research activity reaching a peak in 2025. This sustained growth over the past 5 years indicates that tourists’ emotional experiences have evolved into a mature research domain in destination studies.

**Figure 4:**
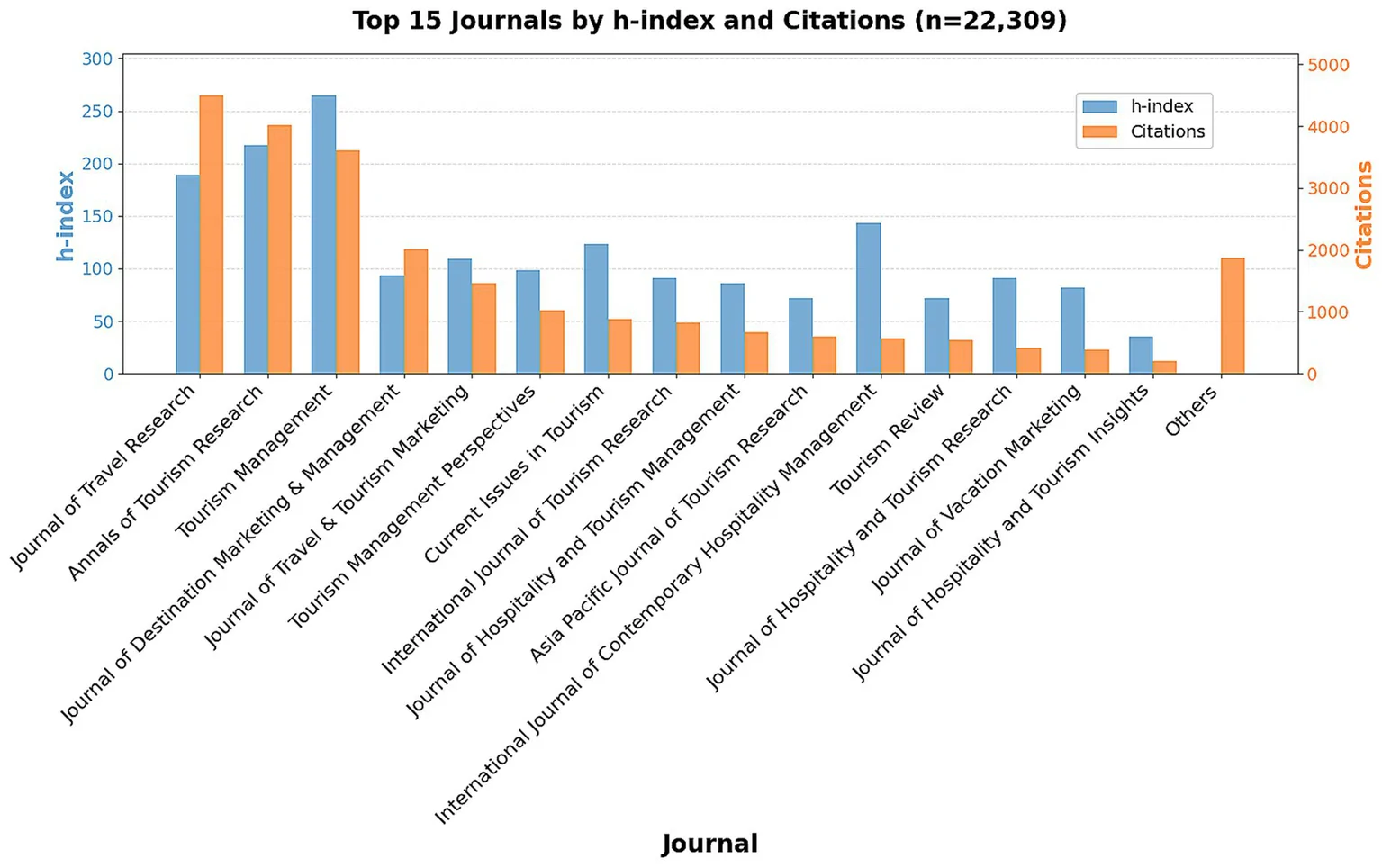
H-index and citation distribution of the top contributing journals (*n* = 22,309).

**Figure 5:**
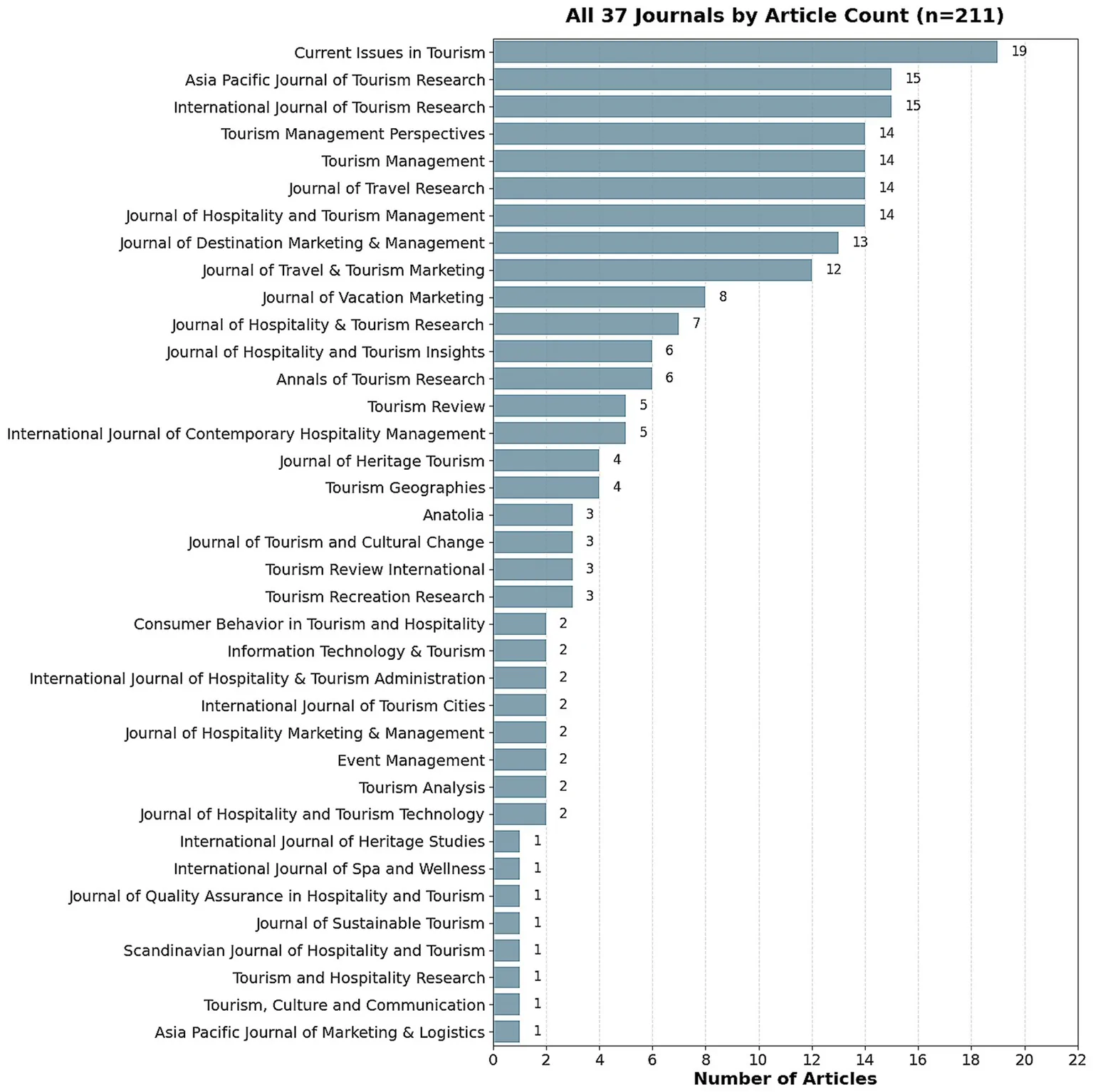
Distribution of articles across journals included in the review (*n* = 211).

**Figure 6:**
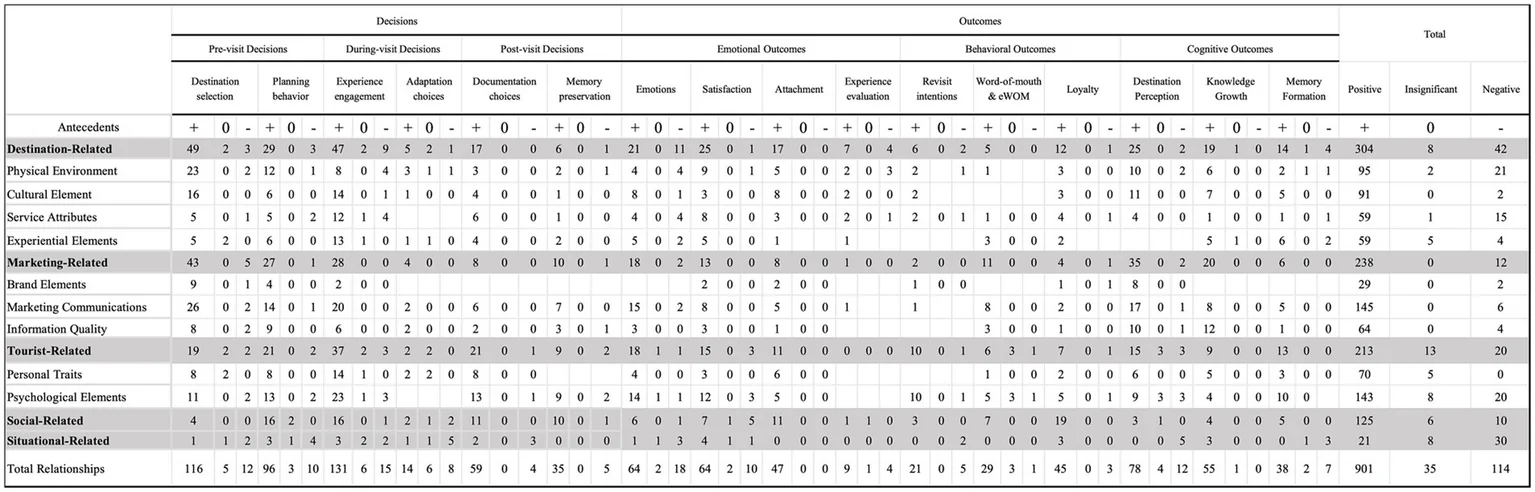
Relationship map of antecedents, decisions, and consequences of tourist emotional experience on destination research. + ‘Positive’, 0 ‘Significant’, and – ‘Negative.

**Figure 7:**
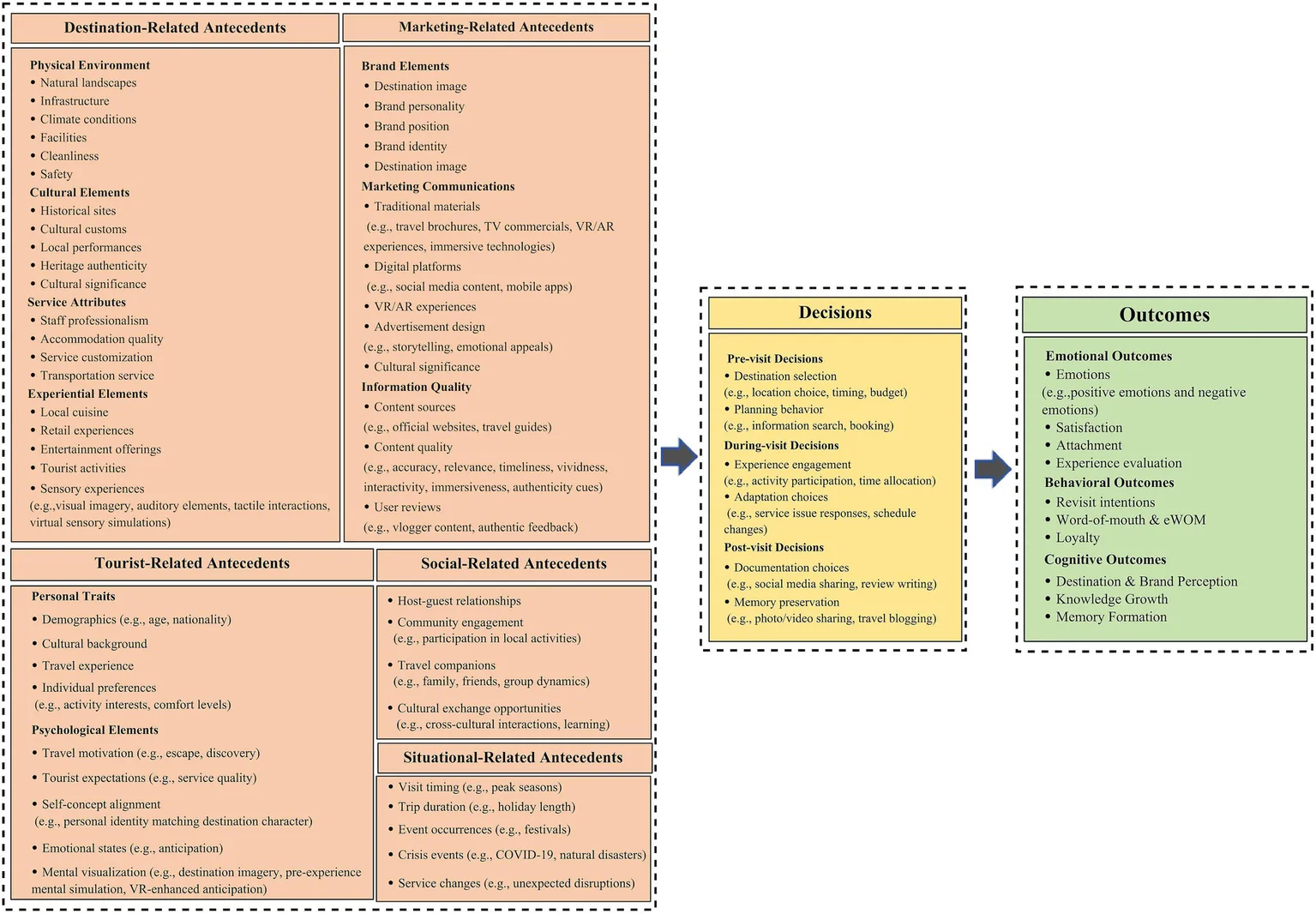
The ADO framework.

The 211 articles have accumulated 22,309 citations (see Figure 4) with the 10 most-cited articles demonstrating particularly strong influence (see Table 1). Among them, Del Bosque and Martín’s (2008) cognitive–affective satisfaction model ranks first, receiving 1,816 citations and followed by Goossens (2000) seminal work on tourism information and pleasure motivation with 1,541 citations. Notably, Prayag et al. (2017) exhibits the highest annual citation rate at 168.5 citations per year, indicating its rapid and sustained academic impact. Regarding publication outlets, the analysis reveals a broad distribution across 37 prestigious journals in tourism research (see Figure 5). Particularly, Current Issues in Tourism leads in publication volume with 19 articles, whereas Journal of Travel Research demonstrates the greatest citation impact, accounting for 5,493 citations. In terms of journal quality, a substantial proportion of the articles were published in top-tier outlets, with 65% appearing in ABDC A-ranked journals (*n* = 137) and an additional 16.5% in A*-ranked journals (*n* = 35). Building on these bibliometric insights, the review synthesizes existing knowledge using the ADO framework. Across the 211 studies, 67 key constructs were identified, including 51 antecedents, 6 decisions, and 10 outcomes. A detailed overview of these constructs can be found in Figure 6 and their interconnections based on the ADO framework are shown in Figure 7.

**Table 1:** Ten most cited articles in the review.

Rank	Title	Author(s)	Total citations (*n* = 8,866)	Citations per year(*n* = 680.6)
1	Tourist satisfaction a cognitive-affective model	Del Bosque and Martín’s (2008)	1816	106.82
2	Tourism information and pleasure motivation	Goossens (2000)	1,541	60.56
3	Understanding the Relationships between Tourists’ Emotional Experiences, Perceived Overall Image, Satisfaction, and Intention to Recommend	Prayag et al. (2017)	1,348	168.5
4	Measuring tourists’ emotional experiences toward hedonic holiday destinations	Hosany and Gilbert (2010)	1,207	80.47
5	The role of tourists’ emotional experiences and satisfaction in understanding behavioral intentions	Prayag et al. (2013)	913	71.7
6	Tourist Perceived Value, Relationship to Satisfaction, and Behavioral Intentions: The Example of the Croatian Tourist Destination Dubrovnik	Pandža Bajs (2015)	767	76.7
7	The Mediating Effect of Place Attachment on the Relationship between Festival Satisfaction and Loyalty to the Festival Hosting Destination	Lee et al. (2012)	701	53.92
8	Exploring user acceptance of 3D virtual worlds in travel and tourism marketing	Huang et al. (2013)	633	52.75
9	Linking travel motivation, tourist self-image and destination brand personality	Murphy et al. (2007)	514	28.56
10	Customer delight from theme park experiences. The Antecedents of Delight based on Cognitive Appraisal Theory	Ma et al. (2013)	487	40.58

Citations per year = Total citations ÷ Current year (2025) minus year of publishing.

### 3.1 Antecedents

In general, antecedents are defined as factors, conditions, events, or variables that occur prior to and influence the outcome of a specific phenomenon, behavior, or decision (Paul and Benito, 2018). The analysis identifies 51 antecedents and grouped into five categories namely destination-related, marketing-related, tourist-related, social-related, and situational-related. Following Kahiya’s (2018) voting method, each reported construct association is treated as one vote rather than being weighted by the number of studies, 901 relationships were identified across these five categories. These relationships collectively illustrate how diverse environmental stimuli and tourists’ psychological factors shape tourists’ emotional experiences and, in turn, influence decision-making processes and behavioral outcomes. The following sections examine these antecedents and their interrelationships within the ADO structure.

#### 3.1.1 Destination-related antecedents

Destination-related antecedents are central environmental stimuli in tourism contexts, including physical environment, cultural elements, service attributes, and experiential components. Together, these dimensions shape the immediate and embodied contexts in which emotional experiences arise. In total, destination-related antecedents received 304 positive votes, with 42 negative and 8 insignificant votes highlighting areas for improvement. Most positive votes are concentrated in the pre-visit and during-visit stages, particularly in destination selection and on-site experience, whereas substantially fewer votes appear in the post-visit stage. This distribution suggests that destination-related antecedents primarily shape expectation formation and real-time experiences, but play a more limited role in post-visit reflection.

##### 3.1.1.1 Physical environment

The physical environment, including natural landscapes, infrastructure, climate, facilities, cleanliness, and safety, serves as a primary stimulus that triggers and shapes tourists’ emotional responses, receiving 95 positive, 21 negative, and 2 neutral votes. Findings indicate that before the trip, factors like natural scenery and infrastructure influence destination choices and planning (Kil et al., 2012; Kim, 2022). Upon arrival, well-maintained facilities and climate enhance emotional engagement and relaxation (Wu and Zhao, 2023; Yitao et al., 2024). Cleanliness and safety also contribute to comfort, loyalty, and advocacy (Elbaz et al., 2023). Negative feedback highlights issues like poor infrastructure and overcrowding will diminish experience quality (Mashkov and Shoval, 2025) suggesting that DMOs should incorporate smart technologies (e.g., AR/VR) as extensions of the physical environment to reshape satisfied emotional experiences through digitally mediated stimuli (Genc et al., 2023).

##### 3.1.1.2 Cultural elements

Cultural factors, including historical sites, traditions, performances, and heritage authenticity, function as symbolic environmental stimuli that evoke meaningful emotional interpretations and connections, receiving 91 positive and 2 negative votes. Before visiting, interest in history and culture influences destination choice (Crespi-Vallbona et al., 2023). During the visiting stage, these elements strongly impact tourist engagement through traditional performances that deepen tourists’ emotional connections (Peng et al., 2023). During the post-visit stage, the perception of emotional experiences will influence tourists’ documenting behavior through photos, videos, or stories, as well as memory preservation and tourists’ emotional connections to a destination (Domínguez-Quintero et al., 2019). These practices enhance attachment and satisfaction (Prayag et al., 2013; Keskin et al., 2024) while shaping destination perceptions and increasing knowledge (Hosseini et al., 2024; Lee et al., 2025). However, concerns arise regarding the risk of misrepresenting authenticity or over-commercializing cultural experiences (Tian et al., 2024) with artificial alterations potentially reducing emotional satisfaction and attachment (Cho and Frochot, 2025). Therefore, future research should focus on preserving cultural authenticity and fostering sustainable cultural interactions to maintain destination competitiveness.

##### 3.1.1.3 Service attributes

Findings show that service-related factors, including staff professionalism, accommodation quality, and transport services, function as interactive environmental stimuli that shape tourists’ emotional experiences through human-environment interactions, receiving 59 positive and 15 negative votes. Reliable service attributes such as high quality accommodation, accessible transportation information, and flexible service options are particularly important before the visit as they support trip planning and reduce uncertainty (Pandža Bajs, 2015). During the visit, professional staff interactions and personalized services contribute significantly to deeper engagement and stronger identification with the destination (Su et al., 2018). Flexible itineraries further allow destinations to respond to diverse visitor needs, which enhances satisfaction and strengthens loyalty over time (Al-Msallam, 2020). In the post visit stage, service quality continues to shape how tourists recall, share, and interpret their experiences, influencing memory formation, word of mouth communication, and intentions to revisit, all of which contribute to destination reputation (Vajpai et al., 2025). However, service failures, poor professionalism, or inflexibility undermine satisfaction and attachment (Ma et al., 2022), and expectation mismatches further weaken loyalty (Chang et al., 2025). Therefore, this review suggests that DMOs should explore emerging technologies as digitally mediated environmental stimuli in their service delivery to address these challenges. For instance, applying artificial intelligence for personalized recommendations (Hosseini et al., 2025), machine learning to predict customer preferences, or automation of service tools to increase service efficiency and reducing errors (Dutta and Dixit, 2025). Overall, these technologies seem to add to customization, simplify operations, and increase capability to better meet the needs of tourists, thereby increasing overall customer satisfaction and loyalty (Ferdowsi and Hosseinzadeh, 2025).

##### 3.1.1.4 Experiential elements

In total, five experiential related antecedents, including local cuisine, retail experiences, entertainment offerings, tourist activities, and sensory experiences, were identified, receiving 59 positive votes, 5 insignificant evaluations, and 4 negative votes. Collectively, these elements can be understood as multi-sensory environmental stimuli that shape tourists’ emotional experiences. Recent studies indicate that food-related activities, entertainment offerings, and sensory environments, play an important role in shaping tourists’ experiences (Kim et al., 2025). Rather than acting at isolated stages of the journey, these elements work together as interconnected dimensions that jointly stimulate emotional engagement and cognitive evaluation. Furthermore, recent literature highlighted that immersive and culturally embedded experiences, such as film to show destination fascination, play an important role early in the tourist journey by shaping expectations and emotional responses that draw visitors into the experience (Veenus et al., 2025). During the visit, interactive offerings further influence tourists’ decisions by deepening engagement and intensifying emotional involvement across different experiential touchpoints (Vajpai et al., 2025). As these emotions become stronger, dense sensory environments encourage tourists to record and share their experiences through photos and videos, allowing the value of the experience to continue beyond the time spent at the destination (Cho and Frochot, 2025).

#### 3.1.2 Marketing-related antecedents

Marketing-related antecedents reflect a distinct category of mediated environmental stimuli that extend emotional experience formation beyond the physical destination. This review identifies three primary dimensions: brand elements, marketing communications (also referred to as destination communication), and information quality. Collectively, these factors received 238 positive votes, with only 12 negative votes and no insignificant votes. Positive votes are concentrated in the pre- and during-visit stages (70 votes and 32 votes respectively) with fewer in the post-visit stage. This suggests that marketing-related factors primarily shape expectations and real-time experiences rather than post-visit reflection. Among them, destination communications have the greatest impact, underscoring the role of storytelling, emotional appeals, and digital platforms like social media and AR/VR. Information quality comes next, emphasizing the importance of accurate, relevant, and timely information in guiding decision-making. Brand elements, meanwhile, play a crucial role in forming tourists’ initial perceptions and mental images of destinations. Importantly, technological advancements have transformed marketing-related stimuli from traditional one-way information transmission into interactive and immersive environments. Through user-generated content, social media engagement, and digital experience design, tourists are no longer passive recipients but active co-creators of emotionally meaningful experiences. This shift further reinforces the role of hybrid (physical-digital) environments in shaping tourist emotional experiences within contemporary destination contexts.

##### 3.1.2.1 Brand elements

The review reveals that brand-related antecedents, including destination image, personality, positioning, and identity, function as symbolic environmental stimuli. These elements shape anticipatory emotions and cognitive representations of destinations and predominantly influences the pre-visit stage of the tourist journey (Letheren et al., 2017; Heredia-Carroza et al., 2025). At this stage, these elements demonstrate a strong effect on shaping tourists’ initial preferences, perceptions, and trust in the destination. However, their influence weakens considerably during the visit and post-visit stages, indicating a gap in brand continuity across the complete travel experience. Regarding outcomes, brand elements exert a substantial effect on cognitive outcomes, particularly in shaping destination perception and tourists’ mental models of the place (Domínguez-Quintero et al., 2019). In contrast, their influence on emotional and behavioral outcomes, such as satisfaction and word-of-mouth, appears moderate. This pattern suggests that while brand elements strongly shape how tourists think about and understand a destination, their capacity to drive affective responses and advocacy behaviors remains underdeveloped. DMOs should therefore strengthen branding throughout the entire visitor journey, creating consistent emotional brand experiences that bridge pre-trip expectations with in-destination touchpoints and post-visit memories to enhance cognitive frameworks and word-of-mouth advocacy.

##### 3.1.2.2 Marketing communications

Marketing communications reflect dynamic and interactive environmental stimuli that actively trigger, amplify, and regulate tourists’ emotional responses across the travel journey. This review identified destination marketing communication related antecedents ranging from traditional media (e.g., brochures, TV commercials) to digital platforms (e.g., social media, mobile apps), VR/AR experiences and advertisement design (e.g., storytelling, emotional appeals). They are critical in shaping tourist emotional experience and behaviors, especially during the pre-visit and on-site stages of the tourist journey. This category received 145 positive votes and 6 negative votes, demonstrating their effectiveness in enhancing tourist experiences. In the pre-visit stage, digital content such as AI-generated hyper-personalized images (Egger and Yu, 2025), effective destination visuals (Alamäki et al., 2023), and VR/AR experiences trigger on-site visit intentions through authenticity perception and emotional resonance (Li et al., 2025). During the visit, immersive technologies increase tourist engagement but also produce mixed effects. While AI-driven tools enhance personalization and emotional connection, they may simultaneously raise privacy concerns and feelings of cultural disconnection (Hosseini et al., 2025). Interestingly, recent evidence suggests that emotional fluctuation, rather than consistently positive affect, is a stronger predictor of tourist well-being, highlighting the value of designing “rhythmic emotional trajectories” instead of uniformly positive experiences (Yao et al., 2025). Overall, digital content bridges online engagement to destination choice through telepresence and flow states (Ferdowsi and Hosseinzadeh, 2025). Those advanced communication strategy generate measurable outcomes. Cognitively, integrated storytelling enhances destination knowledge (Kim and Youn, 2017). Emotionally, short videos foster brand loyalty (Li and Zhang, 2023), while VR triggers stronger reactions and satisfaction than traditional formats (Fan et al., 2022). Moreover, content emphasizing distinctiveness, authenticity, and enjoyment effectively converts engagement into eWOM (Yin et al., 2024). Building on these findings, DMOs should deploy immersive storytelling (VR/AR, AI-personalized content) during pre-visit to build anticipation; utilize interactive tools on-site to sustain engagement; and facilitate shareable experiences post-visit to encourage advocacy. However, effectiveness requires balancing technological innovation with authenticity and privacy concerns.

##### 3.1.2.3 Information quality

Information quality including reliable content sources (e.g., official websites and travel guides), content accuracy, relevance, timeliness, and user-generated reviews. This category received 64 positive and 4 negative votes and primarily influences decision-making in the pre-visit stage. Findings show that high-quality, emotion-driven tourism information (e.g., tourism short videos) significantly shapes destination selection and trip planning (Liu et al., 2025). Similarly, visually rich storytelling (e.g., YouTube travel vlogs) can enhance both emotional and cognitive engagement, strengthening destination memorability (Mirzamurodova Kizi et al., 2025). Research further indicates that source credibility, informational usefulness, entertainment value, escapism, and self-congruence drive word-of-mouth intentions, with credibility exerting the strongest effect on sharing behavior (Cheng et al., 2020). When destinations provide accurate and authentic information that aligns with tourists’ actual experiences, satisfaction increases and positive electronic word-of-mouth becomes more likely (Li et al., 2025).

#### 3.1.3 Tourist-related antecedents

Tourist-related antecedents divide into personal traits and psychological elements, receiving 213 positive, 20 negative, and 13 insignificant votes. From an environmental psychology perspective, these antecedents represent person-side mechanisms that shape how individuals perceive, interpret, and emotionally respond to environmental stimuli across the tourism experience (Gifford, 2014). Among them, psychological elements had the highest impact, followed by personal traits, highlighting the central role of tourists’ personal and psychological characteristics in shaping emotional experiences. A stage-based breakdown shows that positive votes are concentrated in the pre- and during-visit stages (77 votes and 30 votes respectively), with fewer in the post-visit stage (30 votes). This suggests that tourist-related antecedents primarily influence expectation formation and real-time experiences, while playing a less central role in post-visit reflection. This pattern also indicates that internal factors are particularly critical for shaping immediate emotional engagement during the visit.

##### 3.1.3.1 Personal traits

Personal traits received 70 positive votes and no negative ones across studies. Research shows that characteristics such as demographics, cultural background, travel history, and personal preferences consistently influence the tourist journey. In the pre-visit stage, cultural background, prior knowledge, and risk perception shape destination choice, with lower psychological distance and sufficient knowledge strengthening emotional connection and travel intention (Li et al., 2024). Risk concerns also prompt protective behaviors that vary by nationality and gender (Wang et al., 2024). During visits, personality, emotional sensitivity, and cultural values affect engagement depth with emotionally sensitive tourists exhibit stronger responses at dark tourism sites, while curiosity enhances cognitive engagement (Yan et al., 2016). Cultural values further guide behavior, for instance, findings show that collectivist Chinese tourists emphasizing societal impact and South Koreans prioritizing group health (Wang et al., 2024). During post-visit stage, personal traits strongly influence documentation behaviors, as positive emotions encourage sharing whereas negative emotions lead to dissatisfaction (Zhou et al., 2020). Overall, personal traits shape tourism outcomes across emotional, cognitive, and behavioral dimensions. Emotionally, they contribute to destination brand love, nostalgia, and place attachment (Aro et al., 2018; Shi et al., 2021; Steriopoulos et al., 2024). Cognitively, cultural background affects experience interpretation through imagery formation (Ghosh and Sarkar, 2016), knowledge growth (Li et al., 2024), and memory formation (Guleria et al., 2024). Behaviorally, these antecedents contribute to loyalty development through enhanced destination trust (Elbaz et al., 2023) and word-of-mouth (Zhou et al., 2020).

##### 3.1.3.2 Psychological elements

Psychological elements including antecedents like travel motivation, expectations, self-concept alignment, emotional states, and mental imagery. This category received 143 positive, 8 insignificant, and 20 negative votes. In the pre-visit stage, motivation and emotional anticipation drive destination choice, with anticipated feelings showing a significant influence (Filieri et al., 2021). While earlier studies treated travel motivation as relatively stable (Prayag et al., 2017), technology-mediated tools now introduces dynamic emotional anticipation, where emotional fluctuation emerges as a predictor, and tourists actively select flexible routes to optimize well-being through real-time adjustment (Yao et al., 2025). During visits, emotional states mediate destination interactions, with positive emotions enhancing engagement and negative ones prompting avoidance (Huseynov et al., 2020). However, digital contexts reveal an evolving “emotional paradox” whereas earlier studies emphasized arousal’s positive effects (Ito et al., 2023), arousal can now negatively impact intentions when technology-mediated interactions create stress through information overload, forced engagement, or loss of perceived control (Aroa et al., 2025). During the post-visit, psychological elements especially shape documentation, photographing, and nostalgia, creating emotional connections (González-Rodríguez et al., 2020). Notably, the relationship between negative emotions and behavioral intentions has reversed. While unmet expectations traditionally viewed to diminish satisfaction and loyalty (Kim et al., 2022), post-travel negative emotions (e.g., missing the place) can now positively predict revisit intentions (Wang et al., 2025), suggesting technology enables new forms of emotional bonding.

Overall, psychological factors influence emotional, cognitive, and behavioral outcomes. They enhance satisfaction, memory formation, and destination attachment (Prayag et al., 2017), evoke nostalgia through self-congruity (Dai et al., 2025), and drive revisit intentions, word-of-mouth, and loyalty (Huseynov et al., 2020), while deepening understanding and lasting impressions (Filieri et al., 2021). However, cultural tensions persist, indicating the need for improved service quality, cultural sensitivity, and communication alignment (González-Rodríguez et al., 2020). DMOs should therefore manage digital anticipation, on-site emotions, and post-visit emotional continuity, while future research should examine how technology transforms psychological factors into dynamic, co-created processes.

#### 3.1.4 Social-related antecedents

Host-guest relationships, community involvement, travel companions, and cultural exchanges were identified as social-related antecedents. They received 125 positive, 10 negative, 6 insignificant votes. These antecedents capture the social dimensions of environmental stimuli, where interpersonal interactions and shared interpretations influence tourists’ emotional experiences throughout the travel journey. They emerged before travel through anticipated interactions and expectations of community engagement (Cao et al., 2023), during the visit through meaningful interactions with locals and service personnel, and after the visit through memory documentation and sharing behaviors (Şahin and Güzel, 2020). Research reveals that tourist-employee interactions exert stronger influence on place identity formation compared to tourist-resident interactions, suggesting the critical role of service personnel in shaping destination attachment (Das et al., 2025). These social interactions underpin authentic tourism experiences and foster emotional attachment, satisfaction, loyalty, and word-of-mouth advocacy, while also enhancing cultural knowledge and destination image (Fan et al., 2023; Kastenholz et al., 2020; Christou et al., 2018; Min et al., 2020). However, negative social encounters, such as cultural misunderstandings, service failures, unmet interaction expectations, and aggressive selling practices can undermine experience quality and destination appeal, particularly in cross-cultural contexts (Farhat and Chaney, 2021; Kim, 2022; Vajpai et al., 2025). Accordingly, DMOs should therefore prioritize providing positive and authentic host-guest interactions with cultural orientation for visitors, training local communities in cross-cultural communication, and designing genuine interaction opportunities that feel natural rather than forced.

#### 3.1.5 Situational-related antecedents

Situational-related antecedents have mixed effects on tourists’ emotional experiences with 21 positive, 8 insignificant, 30 negative votes received. They include factors such as visit timing, trip duration, event occurrences, crisis situations, and unexpected service disruptions. These factors can be understood as context-dependent environmental stimuli that emerge from specific situations and disruptions, shaping tourists’ emotional responses under changing conditions. Across different stages of the trip, favorable situational cues, particularly appropriate timing and planned events shape destination choice and trip planning. They also enhance on-site experiences through festivals and cultural activities (Goossens, 2000; Lee et al., 2012). After the trip, positive factors like favorable weather, peak season visits, and engaging experiences encourage tourists to document their journey and preserve memories. These elements lead to greater satisfaction and stronger connections to the destination, inspiring loyalty, positive word-of-mouth, and enhancing destination perception while deepening knowledge (Yan and Halpenny, 2022; Şahin and Güzel, 2020; Li et al., 2024). In contrast, risk- and threat-related situational factors, including environmental hazards, health risks, safety concerns, and unethical tourism practices, often trigger threat appraisals, negative moral emotions, and destination avoidance (Wang et al., 2024; Farhat and Chaney, 2021; Fan et al., 2025). It’s worth to notice that such negative events amplified through social media intensify anger, fear, and worry. These emotions often leave lasting memories that reduce revisit intentions and stimulate negative word-of-mouth (Duong et al., 2025; Kim et al., 2024). Building on these findings, we suggest DMOs leverage the use of big data to do real-time sentiment analysis and social media monitoring so as to track public emotional responses during uncertainties. By analyzing vast amounts of user-generated content and online behavioral data, destinations can quickly identify emerging concerns and adjust their communication strategies accordingly.

### 3.2 Decisions

Decisions in tourist emotional experience represent behavioral responses bridging antecedents and outcomes (Paul and Benito, 2018). We structure six key decisions across pre-visit, during-visit, and post-visit phases. The examination of 525 voting relationships reveals distinct patterns of how antecedence influence in these decisions.

#### 3.2.1 Pre-visit decisions

Destination selection and planning behavior are two key pre-visit decisions identified in the review. Findings show that destination selection (133 votes) is primarily driven by destination- and marketing-related antecedents (49 and 43 positive votes), with physical environment and destination communications acting as the strongest drivers. These indicate that such factors shape initial destination imagery through tangible attractiveness and heritage authenticity. Planning behavior (109 votes) reflects a more distributed influence across destination-, marketing-, and tourist-related factors. This distribution implies that the planning stage involves greater information-processing demands, as tourists must integrate diverse external cues with their own cognitive evaluations.

#### 3.2.2 During-visit decisions

During-visit decisions include experience engagement and adaptation choices. Among them, experience engagement receives the strongest influence (153 votes), with destination-related antecedents dominant (47 positive). Cultural and experiential elements show equal strength (14 and 13 positive respectively), indicating balanced tangible-intangible influence. Tourist-related antecedents, particularly psychological elements (23 of 37 positive votes), further indicate that internal processing mechanisms actively filter and interpret these stimuli, reinforcing the role of tourists as co-creators of their experiences. In contrast, adaptation choices (28 votes) show a more balanced distribution across antecedent categories, suggesting that adjustment involves multiple factors, as tourists need to respond to both external conditions and their own expectations and capabilities.

#### 3.2.3 Post-visit decisions

Documentation choices (63 total votes) and memory preservation (40 total votes) represent crucial post-visit decisions that shape long-term destination relationships. Documentation choices are shaped jointly by destination- and tourist-related antecedents. In contrast, memory preservation is more strongly influenced by marketing- and social-related antecedents. This distinction indicates that post-visit processes involve different underlying mechanisms, where environmental factors and individual characteristics guide experience recording and sharing, while longer-term memory construction is more susceptible to external reinforcement from marketing and social contexts.

### 3.3 Outcomes

Outcomes refer to tourists’ evaluative responses following behavioral performance or non-performance (Paul and Benito, 2018). This review classifies the outcomes of tourist emotional experiences into emotional, behavioral, and cognitive categories, which correspond, respectively, to affective, behavioral, and perceptual changes resulting from tourism experiences.

#### 3.3.1 Emotional outcomes

Emotional outcomes represent tourists’ affective responses and evaluations following their tourism experiences (Hosany and Gilbert, 2010; Choi and Choi, 2019). Four outcome constructs with uneven empirical support are identified, namely emotions, satisfaction, attachment, and experience evaluation. Satisfaction (76 relationships) and emotions (84 relationships) receive the strongest support, with most studies reporting positive effects and focusing on the during and post-experience stages (Ito et al., 2023; Fan et al., 2023). Emotions, in contrast, reflect more immediate and subjective affective states that are strongly influenced by individual characteristics, although their context-dependent nature reduces their generalizability. Attachment (47 relationships) reflects longer-term emotional bonds formed through cumulative interactions with destinations, yet it is less frequently examined, particularly in the pre-visit stage. Experience evaluation (9 relationships) remains underexplored, with limited and inconsistent evidence despite its potential to capture more holistic assessments. Overall, emotional outcomes research tends to focus on short-term and positive post-experience responses, while longer-term and mixed or negative emotions receive less attention, indicating a need for more comprehensive and longitudinal approaches.

#### 3.3.2 Behavioral outcomes

Behavioral outcomes refer to tourists’ intended or actual actions following their experiences (Chen and Chen, 2010; Huang et al., 2015). Three behavioral constructs were identified, namely loyalty, word-of-mouth, and revisit intentions. Findings show that loyalty (48 relationships) is strongly driven by social-related factors, underscoring the importance of interpersonal interactions in fostering sustained engagement. Word-of-mouth (33 relationships) is influenced by both destination communication stimuli and social dynamics, showing that both strategic communication and interpersonal interactions encourage tourists to recommend destinations. And revisit intentions (26 relationships) are predominantly shaped by tourist-related factors, emphasizing the role of individual motivations in future visitation decisions. These findings indicate that social interaction is central to fostering loyalty and advocacy, particularly in the post-visit stage, whereas personalized experiences are more important for encouraging revisit intentions.

#### 3.3.3 Cognitive outcomes

Cognitive outcomes reflect changes in tourists’ knowledge and perceptions resulting from tourism experiences (Skavronskaya et al., 2017; Tung and Ritchie, 2011). Three cognitive outcomes were identified, they are destination perception, knowledge growth, and memory formation. Among these, destination perception (94 relationships) receives the strongest support and is mainly shaped by marketing-related and destination-related antecedents. Most evidence focuses on the pre-visit stage, where perceptions are formed through destination communication (e.g., storytelling) and destination attributes (Kim, 2022; Li and Zhang, 2023). Knowledge growth (56 relationships) is influenced by both marketing and destination-based factors. This is mainly observed during the visit stage, where tourism information and on-site learning opportunities jointly contribute to visitors’ understanding (Lee et al., 2025). Memory formation (47 relationships) is primarily shaped by tourist- and destination-related factors. This pattern is more evident in the post-visit stage, where individual traits and destination distinctiveness influence how experiences are remembered (Guleria et al., 2024). These findings highlight the importance of integrating marketing, destination, and personalized factors in shaping cognitive outcomes.

## 4 Where should we be heading?

Building on the synthesis above, this review identifies three key directions for advancing research on tourist emotional experiences through the lens of human-environment interactions in destination contexts. The following subsections examine each area and propose targeted research agendas.

### 4.1 Theorizing emotional triggering and cross-stage transmission mechanisms

Although the ADO-based analysis maps 525 voting relationships between antecedents and decisions, revealing how diverse environmental stimuli influence tourist behavior differently at each stage of the journey. A fundamental theoretical question remains unresolved: through what mechanisms do emotions formed in one stage carry forward to influence responses in subsequent stages? This gap becomes particularly evident when considering the stage-specific patterns identified in the present synthesis? This gap becomes particularly evident when considering the stage-specific patterns identified in the present synthesis.

In the pre-visit stage, tourist responses are primarily shaped by destination- and marketing-related factors, particularly physical environments and destination communications. These external cues contribute to the formation of initial emotional expectations and images of the destination. During the visit, the role of psychological processes becomes more prominent, as tourists’ internal states and subjective evaluations increasingly shape how they interpret environmental stimuli. In the post-visit stage, emotional experiences are influenced by a broader combination of psychological reflection and social interactions, which contribute to memory formation and sharing behaviors. These stage-specific patterns suggest that environmental stimuli do not generate fixed emotional responses but are continuously interpreted across the travel journey. For example, a VR preview encountered before travel not only influences travel intentions but also shapes expectations that may later be confirmed or contradicted by the on-site experience. Similarly, this review shows that negative post-travel emotions, such as “missing the destination,” can strengthen revisit intentions through nostalgia and emotional attachment (Wang et al., 2025). This finding challenges the conventional expectation-disconfirmation framework (Kim et al., 2022) and highlights the need to understand emotional experiences as a process of stage-specific reappraisal rather than as fixed responses.

Although decision-making research has increasingly recognized the importance of temporal dynamics (Moore et al., 2012; Mihai et al., 2023), tourism studies have largely continued to treat antecedents as static factors rather than as stage-dependent stimuli whose emotional effects may change with tourists’ evolving psychological states. Existing research has rarely examined how emotions formed at one stage influence responses at subsequent stages, or how these carry-over effects unfold over the course of the journey (Lin and Nawijn, 2020). Consequently, current understanding remains limited in explaining why the same environmental conditions may generate different emotional responses across journey stages and why expectation-reality discrepancies can sometimes produce stronger emotional reactions than confirmed experiences (Smallman and Moore, 2010; Stylos, 2020).

To address this gap, future research should investigate a stage-specific emotional reappraisal mechanism. Rather than treating each stage of the journey as an isolated episode, the tourist journey can be understood as a continuous process in which tourists continuously reinterpret their emotions in response to evolving experiences. Emotions formed at earlier stages may influence how subsequent experiences are perceived and evaluated. For example, expectations developed before the visit can shape interpretations of on-site environments, while post-visit reflections may further reconstruct earlier experiences through memory and social sharing. This suggests that emotional responses are not fixed outcomes but are continuously updated throughout the journey. Taken together, these dynamics highlight the importance of cross-stage emotional carry-over, whereby emotions experienced at one stage become inputs that shape responses at later stages. However, the mechanisms underlying this process remain unclear, therefore, several research questions were proposed for future research (see Table 2).

**Table 2:** Future research questions.

Major direction	Suggested research questions
Theorizing emotional triggering and cross-stage transmission mechanisms	What cognitive reappraisal processes explain why the same environmental stimulus (e.g., VR immersion or influencer-generated content) generates excitement before travel but leads to stress or emotional overload during the visit? What individual-level factors, such as emotional sensitivity, cultural background, or prior travel experience, shape these outcomes? (H)
	How do emotional carry-over effects operate across different stages of travel journey? Under what conditions does pre-visit anticipation enhance or diminish on-site satisfaction, and through what psychological mechanisms? (H)
	What drives transitions between the pre-visit, during-visit, and post-visit stages, especially when expectations conflict with actual experiences? How do tourists resolve such discrepancies, and how can destinations support adaptive rather than avoidant responses? (H)
	How do post-visit processes, such as memory consolidation, social sharing, and nostalgic reflection reshape the emotional meaning of on-site experiences? Under what conditions can negative emotions be reframed into positive long-term attachment? (H)
	How can destinations strategically design and sequence environmental stimuli, such as AI-driven personalization before travel, sensory design during the visit, and community-based platforms afterward, to create emotionally coherent experiences across the entire tourist journey? (H)
Rethinking technology as hybrid environmental stimuli	Under what conditions do digital environmental stimuli (e.g., AI personalization, VR previews, social media content) function as restorative versus stressful inputs within the SOR framework? How do stimulus characteristics (e.g., authenticity, information load, interactivity, personalization) and individual differences (e.g., technology readiness, cultural background, prior experience) shape these effects? (H)
Rethinking technology as hybrid environmental stimuli	How does congruence between pre-visit digital representations and on-site experiences influence emotional responses across stages? At what point does mismatch lead to cognitive dissonance that overrides hedonic engagement, and how can destinations manage this risk? (H)
	How do hybrid environments, where physical and digital elements coexist (e.g., AR-enhanced heritage sites or gamified experiences), generate emotional outcomes that differ from those produced by either domain alone? What theoretical constructs are needed to capture these emergent effects? (H)
	What kind of theoretical model can explain when and how technology enhances or undermines emotional experiences across different stages of the tourist journey, rather than assuming uniformly positive technological effects? How might such a model integrate perspectives from environmental psychology, cognitive appraisal theory, and human-computer interaction? (H)
	How do algorithm-driven systems such as recommendation engines and social media feeds shape tourists’ anticipatory emotional expectations prior to travel, and what occurs when these algorithmically constructed expectations differ from on-site experiences? (L)
Exploring environmental stressors and emotional resilience	How do different type crisis-related environmental stressors impact tourists’ cognitive evaluations and behavioral decision-making across journey phases? (L)
	What restorative environmental cues and affective communication strategies can destinations use to reduce perceived risk and support tourists’ emotional well-being during crises? (H)
	How do post-crisis environmental recovery efforts and image rebuilding shape tourists’ long-term place attachment and loyalty? (L)
	What specific governance structures and incentive mechanisms enable effective crisis collaboration across stakeholders? How should decision-making authority be distributed among DMOs, local governments, and businesses to ensure consistent and stable environmental cues are communicated to tourists across different crisis phases (e.g., prevention vs. response)? What financial/reputational incentives motivate private enterprises to participate in long-term resilience-building? (L)

H, High-impact research priority; L, Lower-priority or exploratory direction.

### 4.2 Rethinking technology as hybrid environmental stimuli

This synthesis shows that digital environmental stimuli, including social media platforms, virtual and augmented reality, AI-driven personalization tools, and user-generated content, have increasingly shaped tourist emotional experiences across different stages of the travel journey. However, their emotional effects are far from uniform. For example, existing studies suggest that digital stimuli can enhance anticipation and travel intentions in the pre-visit stage (Egger and Yu, 2025), while generating mixed or even negative emotional responses during on-site experiences due to information overload and excessive digital engagement (Hosseini et al., 2025; Aroa et al., 2025). These inconsistencies suggest that the emotional effects of technology depend on the interaction between digital stimuli, situational contexts, and journey stages.

This variability highlights limitations in how existing environmental psychology frameworks, such as the Pleasure-Arousal-Dominance (PAD) model and the Stimulus-Organism-Response (SOR) paradigm (Mehrabian and Russell, 1974), have been applied to tourism contexts. Although these frameworks provide valuable explanations of how environmental stimuli influence emotional responses, they offer limited insight into why the same digital technologies may generate different emotional outcomes depending on journey stage and situational context. More specifically, existing theories provide insufficient explanations of how digital stimuli shape emotions differently before, during, and after travel, how technology can simultaneously enhance engagement and create stress, and how digital and physical environmental cues jointly contribute to tourist emotional experiences.

To address this gap, future research should develop the concept of hybrid stimulus congruence, which refers to the extent to which digital environmental stimuli align with tourists’ emotional expectations and existing psychological schemas across different stages of the journey. Drawing on schema congruity theory (Mandler, 2014) and restorative environment theory (Kaplan, 1995), this perspective suggests that digital stimuli are more likely to generate positive emotional responses when they are consistent with, or moderately discrepant from, tourists’ existing expectations. When digital content accurately reflects the sensory, cultural, and experiential characteristics of a destination, it can reduce uncertainty, strengthen positive anticipation, and facilitate emotional engagement. In contrast, when digital representations create unrealistic expectations, diverge from on-site experiences, or introduce cognitive burdens such as information overload, algorithmic opacity, or privacy concerns (Ito et al., 2023), they may function as stressors that disrupt emotional responses. This perspective also helps explain why the effects of digital stimuli vary across journey stages. Before travel, tourists may be more receptive to idealized digital representations because expectations remain flexible. During the visit, direct experiences become more influential, making mismatches between digital representations and reality more salient. After the trip, digital platforms may reshape emotional memories through selective sharing and social feedback. Building on these insights, several directions for future research are proposed (see Table 2).

### 4.3 Exploring environmental stressors and emotional resilience

The findings indicate crisis management and destination resilience as critical directions for future research particularly given situational factors’ vulnerability with more negative (30) than positive (21) votes. As the global tourism environment grows increasingly complex with frequent disruptions such as natural disasters (Wang et al., 2024), unethical incidents (Fan et al., 2025), and tragedy-induced negative social media (Duong et al., 2025), they act as severe situational stressors. Therefore understanding how such environmental shocks impact tourist decision-making across journey phases becomes urgent, especially considering situational factors’ strong influence on pre-visit planning (26.7% of votes).

Future studies should explore how destinations can combine emotional appeals to maintain tourists’ confidence by using digital platforms that deliver timely and reliable information (Ferdowsi and Hosseinzadeh, 2025). The use of multiple communication channels can help shape emotional responses and reduce perceived risk under uncertain conditions thereby supporting tourist confidence (Duong et al., 2025). Second, future studies should examine how destinations can protect positive emotional outcomes during crisesin contexts characterized by stress, negative emotions, or emotional complexity, as these conditions have been shown to influence satisfaction, happiness, and overall experience evaluation (Wang et al., 2025). Third, greater attention is needed on the long-term influence of crisis responseincluding its effects on destination image, loyalty, and word-of-mouth (Jiang and Garrod, 2025; Cho and Frochot, 2025). These priorities highlight the need for collaboration across destination organizations, local communities, enterprises, and government. Future research should also consider how collaborative approaches contribute to integrated crisis management and enhance destination resilience across prevention, preparation, response, and recovery phases. Based on these gaps, we propose the several research questions in Table 2.

## 5 Conclusion

This systematic review provides the first domain-level synthesis of tourist emotional experience in destination contexts. By reviewing 211 high-quality studies published between 2000 and 2025, we systematically organize the domain around antecedents, decisions, and outcomes. Using the ADO framework, 901 relationships were mapped among 51 antecedents, 6 decision constructs, and 10 outcome constructs. The synthesis reveals how tourist emotional experiences emerge and evolve across the travel journey, offering new insights for destination research and practical guidance for managing tourist emotions.

### 5.1 Theoretical implications

The ADO framework-based review makes several theoretical contributions to tourist emotional experience research. First, this study advances a dynamic, journey-based perspective by showing that tourist emotional experiences are not static responses to isolated stimuli but evolve through continuous appraisal and reinterpretation across the pre-visit, on-site, and post-visit stages. Emotional outcomes such as satisfaction, attachment, and loyalty should therefore not be viewed as independent responses, but as consequences of accumulated emotional experiences throughout the journey. Specifically, emotions are initially shaped by destination attributes and communication cues before travel, further influenced by tourists’ psychological engagement during the visit, and subsequently reshaped through post-visit reflection and social interaction. From an environmental psychology perspective, this temporal process aligns with Gifford’s (2014) transactional view of person-environment interaction, which emphasizes that individuals actively interpret, adapt to, and construct meanings from changing environmental conditions rather than passively responding to fixed stimuli. Applied to the tourist journey, this perspective explains why the same environmental stimulus may generate different emotional responses across stages and why the behavioral implications of emotions may change as tourists progress through the journey.

An important implication of this journey-based perspective is that it challenges the assumption that emotional valence has a consistent relationship with behavioral outcomes across contexts. Expectation-disconfirmation theory (EDT) traditionally suggests that negative evaluations arising from unmet expectations may reduce satisfaction and weaken behavioral intentions such as loyalty and revisit intentions (Oliver, 1980; Spreng et al., 1996). However, this review identifies a more nuanced pattern. Post-travel negative emotions, such as the feeling of “missing the destination,” may sometimes strengthen revisit intentions rather than diminish them (Wang et al., 2025). This suggests that negative emotions do not carry fixed behavioral meanings across journey stages. Instead, emotions experienced after travel may be reinterpreted as longing, nostalgia, or deeper attachment, thereby reinforcing rather than weakening the tourist-destination relationship. This finding extends EDT by showing that the link between emotional valence and behavioral outcomes depends on temporal context and the way emotions are subsequently interpreted. Future research should therefore adopt longitudinal designs and real-time measurement approaches to capture the evolving nature of tourist emotional experiences. Advanced methods such as eye-tracking, EEG, galvanic skin response (GSR) (Lei et al., 2024), sentiment analysis, and machine-learning-based multimodal analytics may help reveal how conscious evaluations and implicit emotional reactions develop before, during, and after travel.

Second, this review highlights crisis management and emotional resilience as an important area for future research. Drawing on environmental psychology, crisis events can be conceptualized as acute environmental stressors that disrupt tourists’ expected experiences, trigger threat appraisals, and influence subsequent emotional and behavioral responses. Future research should integrate stress and coping perspectives (e.g., cognitive appraisal theory; Lazarus, 1991) to examine how tourists perceive and cope with environmental disruptions, and how restorative environmental cues may buffer negative emotional responses during crises. Further research may also explore how destination stakeholders develop collaborative strategies to enhance resilience in times of disruption.

Third, the review reveals how technology has fundamentally reshaped tourist experiences across the entire journey, from AI-based personalization in pre-visit planning, to VR/AR immersion during on-site experiences, and digital platforms for post-trip sharing. These findings extend environmental psychology beyond its traditional focus on physical settings, such as natural landscapes and built environments, to include hybrid environments that integrate physical, sensory, symbolic, and digital stimuli. This shift highlights that digital platforms, virtual reality, and AI-driven personalization systems can also function as environmental stimuli, shaping emotional responses, influencing behavioral intentions, and contributing to the formation of person-place relationships, even in the absence of physical co-presence. However, these technologies often generate mixed emotional effects. For example, AI-driven personalization can heighten privacy anxiety (Hosseini et al., 2025), VR-based immersion may induce stress rather than excitement in certain contexts (Aroa et al., 2025), and algorithm-driven content can create information overload that undermines decision-making. These findings challenge the assumption that digital technologies uniformly enhance tourist experiences. Therefore, this review proposes the concept of hybrid stimulus congruence to explain how the alignment between digital environmental stimuli and tourists’ psychological schemas shapes emotional responses across different journey stages.

### 5.2 Practical implications

Practically, this synthesis offers guidance for DMOs to design stage-specific strategies that shape tourist emotional experiences across the travel journey. The ADO findings show that pre-visit emotions are primarily influenced by destination attributes and communication cues, on-site emotions are shaped by tourists’ psychological engagement with the destination, and post-visit emotions are reinforced through social interactions and sharing activities. These patterns highlight the importance of managing emotional touchpoints in ways that reflect tourists’ evolving needs and experiences across different stages of the journey. Drawing on environmental psychology, such strategies involve the intentional design of physical, sensory, symbolic, and digital stimuli to create meaningful emotional experiences throughout the journey.

In practice, DMOs can strengthen pre-visit engagement by immersive digital previews such as 360°virtual tours, AR-enhanced maps, and themed itinerary generators can help visitors form vivid expectations of the destination (Fan & Deng., 2022). Story-driven content (e.g., storytelling), including short video or destination film can further enhances emotional anticipation before the trip (Ruan et al., 2025; Veenus et al., 2025).

During the visit, emotional immersion can be strengthened through thoughtful service design and environmental cues that support comfort, engagement, and personalization. For instance, DMOs should enhance psychological comfort during visits through real-time sentiment monitoring via social media analytics to detect emerging negative emotions (pollution complaints, overcrowding frustration) and trigger immediate interventions (Duong et al., 2025). Train service personnel as “emotional architects” is another effective strategy, as employee interactions shape place identity more powerfully than resident encounters (Das et al., 2025). Moreover, emotional immersion can be strengthened through gamified tourism experiences that incorporate entertainment value and social interaction, thereby enhancing users’ engagement and perceived usefulness (Malik et al., 2026).

After the visit, emotional connections can be reinforced through strategies that sustain social meaning and encourage reflection (Godovykh and Tasci, 2021). DMOs can create community platforms for ongoing interaction, host online or hybrid cultural events, and send timed reminders that help visitors recall memorable moments (Zhou et al., 2020). Documentation and sharing can be encouraged through auto-generated travel memory videos, visualizations of emotional journeys, digital souvenirs, and structured systems that reward user-generated content (Sykora et al., 2022). These approaches help extend emotional attachment and turn visitors into active contributors to the destination’s narrative. Managing these touchpoints in a phased manner enables DMOs to guide emotional experiences more effectively than with one-size-fits-all approaches.

### 5.3 Limitations

Several limitations of this review should be acknowledged. Despite the comprehensive search strategies across Web of Science and Scopus databases, some relevant studies may have been excluded due to the focus on ABDC-listed journals and English-language publications. In addition, the review’s temporal scope (2000–2025) may have missed earlier foundational works. Furthermore, this review adopts the ADO framework to synthesize existing research, primarily addressing what is currently known in terms of key antecedents, decisions, and outcomes of tourist emotional experiences. However, ADO provides limited insight into how such knowledge has been generated, particularly regarding theoretical foundations, research contexts, and methodological choices. Future reviews could address these limitations by expanding database coverage, including non-English and non-ABDC publications, extending the temporal scope, and integrating alternative frameworks such as TCM (Theory-Context-Method) or TCCM (Theory-Context-Characteristics-Method) framework to complement ADO and better capture the landscape of tourist emotional experiences research. Despite these limitations, this review offers valuable contributions. For academics, it provides a phase-structured understanding of tourist emotional experience across the travel journey and identifies promising avenues for future research. For practitioners, it highlights the importance of managing tourist emotions across different journey stages and offers guidance for enhancing destination competitiveness through emotion-oriented strategies.

## Data Availability

The original contributions presented in the study are included in the article/Supplementary material, further inquiries can be directed to the corresponding author/s.
