# Supplementary material for: From anticipation to memory: a systematic review of tourist emotional experience
Source: Front Psychol. 2026 Aug 7;17:1907118. doi: 10.3389/fpsyg.2026.1907118 (PMC13493304; doi:10.3389/fpsyg.2026.1907118)
Supplement: Supplementary file 1 [file Supplementary_file_1.docx]

**Appendix.** Supplementary material

**Appendix 1.** Search strings and results in WoS and Scopus

As detailed in Section 2 of the manuscript, a comprehensive literature search was conducted using two influential academic databases: Web of Science (WoS) and Scopus. We employed specific search strings to identify relevant publications related to tourist emotional experience in destination marketing. The search covered publications from January 1, 2000, to December 31, 2025, to capture both foundational works and recent developments in the field. Table A1 presents the searching strings and results in WoS and Scopus.

**Table A1**. Searching strings and results in WoS and Scopus.

| **Database** | **Search strings** | **Search results** |
| --- | --- | --- |
| Web of Science (Wos) | TS= (("emotion" OR "emotional experience" OR "emotional response" OR "emotional reaction" OR "emotional connection" OR "emotional engagement" OR "emotional attachment" OR "emotional bond" ) AND ( "destination") AND ( “ tourism”)) AND Hospitality Leisure Sport Tourism (Web of Science Categories) AND Article (Document Types) AND English (Languages) | 307 |
| Scopus | TITLE-ABS-KEY ( "emotion" OR "emotional experience" OR "emotional response" OR "emotional reaction" OR "emotional connection" OR "emotional engagement" OR "emotional attachment" OR "emotional bond" ) AND TITLE-ABS-KEY ( destination AND tourism* ) AND PUBYEAR > 1999 AND PUBYEAR < 2026 AND ( LIMIT-TO ( SRCTYPE , "j" ) ) AND ( LIMIT-TO ( PUBSTAGE , "final" ) ) AND ( LIMIT-TO ( DOCTYPE , "ar" ) ) AND ( LIMIT-TO ( LANGUAGE , "English" ) ) AND ( LIMIT-TO ( SUBJAREA , "BUSI" ) OR LIMIT-TO ( SUBJAREA , "SOCI" ) ) | 696 |

**Appendix 2.** **List of** ABDC journals included in the review based on the 2022 ABDC journal ranking

As detailed in Section 2 of the manuscript, this study utilized the Australian Business Deans Council (ABDC) Tourism Journal Quality List (2022 edition) as a key criterion to ensure all included articles were from high-quality, credible sources. The ABDC list is a widely recognized benchmark for academic journal quality, particularly in business research, and includes many journals that are also featured in other prestigious ranking indices such as the Social Sciences Citation Index (SSCI). Table A2 presents ABDC journals included in the review based on the 2022 ABDC journal ranking.

**Table A2.** List of ABDC journals included in the review based on the 2022 ABDC journal ranking

| **No.** | **Journal title** | **Publisher** | **ISSN** | **ISSN online** | **Year** | **Rating** |
| --- | --- | --- | --- | --- | --- | --- |
| 1 | Tourism Management Perspectives | Elsevier | 2211-9736 | 2211-9744 | 2012 | A |
| 2 | Asia Pacific Journal of Tourism Research | Taylor & Francis Online | 1094-1665 | 1741-6507 | 1996 | A |
| 3 | Tourism Management | Elsevier | 0261-5177 | 1879-3193 | 1983 | A* |
| 4 | Journal of Travel Research | Sage Publications | 0047-2875 | 1552-6763 | 1962 | A* |
| 5 | Journal of Destination Marketing & Management | Elsevier | 2212-571X | 2212-5752 | 2012 | A |
| 6 | Current Issues in Tourism | Taylor & Francis Online | 1368-3500 | 1747-7603 | 1998 | A |
| 7 | International Journal of Tourism Research | Wiley-Blackwell Publishing | 1099-2340 | 1522-1970 | 1998 | A |
| 8 | Journal of Travel & Tourism Marketing | Taylor & Francis Online | 1054-8408 | 1540-7306 | 1992 | A |
| 9 | Journal of Hospitality and Tourism Management | Elsevier | 1447-6770 | 1839-5260 | 1994 | A |
| 10 | Journal of Vacation Marketing | Sage Publications | 1356-7667 | 1479-1870 | 1995 | A |
| 11 | Journal of Hospitality and Tourism Insights | Emerald Group Publishing | 2514-9792 | 2514-9806 | 2018 | C |
| 12 | Annals of Tourism Research | Elsevier | 0160-7383 | 1873-7722 | 1973 | A* |
| 13 | Tourism Review | Emerald Group Publishing | 1660-5373 | 1759-8451 | 2001 | B |
| 14 | International Journal of Contemporary Hospitality Management | Emerald Group Publishing | 0959-6119 | 1757-1049 | 1989 | A |
| 15 | Journal of Heritage Tourism | Taylor & Francis Online | 1743-873X | 1747-6331 | 2006 | B |
| 16 | Journal of Hospitality and Tourism Research | Sage Publications | 1096-3480 | 1557-7554 | 1976 | A |
| 17 | Tourism Geographies | Taylor & Francis Online | 1461-6688 | 1470-1340 | 1999 | A |
| 18 | Anatolia | Taylor & Francis Online | 1303-2917 | 2156-6909 | 1990 | B |
| 19 | Journal of Tourism and Cultural Change | Taylor & Francis Online | 1476-6825 | 1747-7654 | 2003 | B |
| 20 | Tourism Review International | Cognizant Communication Corporation | 1544-2721 | 1943-4421 | 2000 | C |
| 21 | Consumer Behavior in Tourism and Hospitality | Emerald Group Publishing | 2752-6666 | 2752-6674 | 2007 | B |
| 22 | International Journal of Tourism Cities | Emerald Group Publishing | 2056-5607 | 2056-5615 | 2015 | C |
| 23 | Journal of Hospitality Marketing and Management | Taylor & Francis Online | 1936-8623 | 1936-8631 | 2009 | A |
| 24 | Information Technology & Tourism | Springer International Publishing | 1098-3058 | 1943-4294 | 1998 | B |
| 25 | International Journal of Hospitality and Tourism Administration | Taylor & Francis Online | 1525-6480 | 1525-6499 | 2000 | B |
| 26 | International Journal of Heritage Studies | Taylor & Francis Online | 1352-7258 | 1470-3610 | 1994 | B |
| 27 | Event Management | Cognizant Communication Corporation | 1525-9951 | 1943-4308 | 1993 | A |
| 28 | International Journal of Spa and Wellness | Taylor & Francis Online | 2472-1735 | 2472-1743 | 2018 | C |
| 29 | Journal of Quality Assurance in Hospitality &Tourism | Taylor & Francis Online | 1528-008X | 1528-0098 | 2000 | B |
| 30 | Journal of Sustainable Tourism | Taylor & Francis Online | 0966-9582 | 1747-7646 | 1993 | A* |
| 31 | Scandinavian Journal of Hospitality and Tourism | Taylor & Francis Online | 1502-2250 | 1502-2269 | 2001 | A |
| 32 | Tourism Analysis | Cognizant Communication Corporation | 1083-5423 | 1943-3999 | 1996 | A |
| 33 | Tourism and Hospitality Research | Sage Publications | 1467-3584 | 1742-9692 | 1999 | B |
| 34 | Tourism Recreation Research | Taylor & Francis Online | 0250-8281 | 2320-0308 | 1976 | A |
| 35 | Tourism, Culture and Communication | Cognizant Communication Corporation | 1098-304X | 1943-4146 | 1998 | C |
| 36 | Asia Pacific Journal of Marketing & Logistics | Emerald Group Publishing | 1355-5855 | 1758-4248 | 1993 | A |
| 37 | Journal of Hospitality and Tourism Technology | Emerald Group Publishing | 1757-9880 | 1757-9899 | 2010 | B |

**Appendix 3.** List of articles included in the review

As detailed in Section 2 of the manuscript, we employed the PRISMA protocol to systematically identify and select relevant articles. The initial search in Web of Science and Scopus databases yielded 1003 potentially relevant articles (307 from Web of Science and 696 from Scopus). After removing 160 duplicates, the remaining 843 articles underwent screening based on ABDC Tourism Journal Quality List and relevance to the study topic, resulting in 360 articles for full-text review. Following quality assessment using the Quality Assessment with Diverse Studies (QuADS) instrument, a final sample of 211 high-quality articles published between 2000 and 2025 were included in the systematic review. The complete list of these articles is presented below.

1. Adie, B. A., & de Bernardi, C. (2023). “Oh my god what is happening?”: Historic second home communities and post-disaster nostalgia. *Journal of Heritage Tourism*, *18*(2), 1–12. <https://doi.org/10.1080/1743873x.2020.1828429>
2. Akgün, A. E., Senturk, H. A., Keskin, H., & Onal, I. (2020). The relationships among nostalgic emotion, destination images and tourist behaviors: An empirical study of Istanbul. *Journal of Destination Marketing & Management*, *16*(1). <https://doi.org/10.1016/j.jdmm.2019.03.009>
3. Al‐Msallam, S. (2020). The impact of tourists’ emotions on satisfaction and destination loyalty – an integrative moderated mediation model: tourists’ experience in Switzerland. *Journal of Hospitality and Tourism Insights*, *3*(5), 509–528. <https://doi.org/10.1108/jhti-11-2019-0126>
4. Alamäki, A., Rhee, C., Suomala, J., Kaski, T., & Kauttonen, J. (2023). Creating effective visuals for destination marketing videos: Scenery vs people. *Journal of Vacation Marketing*, *29*(1), 135676672210811. <https://doi.org/10.1177/13567667221081147>
5. Anam Fatma, & Bhatt, V. (2024). Sculpting the feelings: influence of immersive technology on responsible travel. *International Journal of Contemporary Hospitality Management, 36(11)*. https://doi.org/10.1108/ijchm-09-2023-1491
6. Aro, K., Suomi, K., & Saraniemi, S. (2018). Antecedents and consequences of destination brand love — A case study from Finnish Lapland. *Tourism Management*, *67*, 71–81. <https://doi.org/10.1016/j.tourman.2018.01.003>
7. Aroa Costa-Feito, Rodríguez-Santos, C., González-Fernández, A. M., & Santos. (2025). The emotional paradox of short promotional videos in urban tourism: cognitive and affective responses in decision-making under stress or joy. *Journal of Travel & Tourism Marketing, 42(5),* 708–723. https://doi.org/10.1080/10548408.2025.2505890
8. Ayush Guleria, Joshi, R., & Adil, M. (2024). The impact of memorable tourism experiences on customer-based destination brand equity: the mediating role of destination attachment and overall satisfaction. *Journal of Hospitality and Tourism Insights*. <https://doi.org/10.1108/jhti-03-2023-0220>
9. Beckman, E., Whaley, J. E., & Kim, Y.-K. (2017). Motivations and experiences of whitewater rafting tourists on the Ocoee River, USA. *International Journal of Tourism Research*, *19*(2), 257–267. <https://doi.org/10.1002/jtr.2109>
10. Bhardwaj, H., & Sharma, D. (2025). Softening the dark: the role of cute aggression in shaping travel intentions toward dark tourism destinations. *Asia Pacific Journal of Tourism Research,* 1–31. https://doi.org/10.1080/10941665.2025.2574091
11. Bi, N., Yin, C.-Y., & Chen, Y. (2020). A bittersweet experience! The effect of mixed emotions on business tourists’ revisit intentions. *Journal of Travel & Tourism Marketing*, *37*(6), 695–710. <https://doi.org/10.1080/10548408.2020.1795047>
12. Brodien Hapairai, P. M., Walters, G., & Li, S. (2018). The effectiveness of ad-induced emotion in reducing tourist risk perceptions towards politically unstable destinations. *Tourism Recreation Research*, *43*(4), 483–496. <https://doi.org/10.1080/02508281.2018.1470737>
13. Cai, Y., Gong, Z., Yang, Y., Xiao, Y., & Zhou, J. (2025). Embodied interaction and cultural Involvement: How familiarity and challenge shape tourism rituals. *Journal of Hospitality and Tourism Management,* 65, 101335–101335. <https://doi.org/10.1016/j.jhtm.2025.101335>
14. Cao, L., Xie, D., & Qu, Y. (2023). A process perspective on experience co-creation: How pre-trip involvement prompts destination loyalty. *Tourism Management*, *96*, 104711. <https://doi.org/10.1016/j.tourman.2022.104711>
15. Carden, A. R. (2006). Sign of the times: An examination of the self-concept and emotional motivation of travelers on route 66 (1926-Present). *Journal of Travel & Tourism Marketing*, *20*(2), 51–61. <https://doi.org/10.1300/j073v20n02_04>
16. Chan, B. (2007). Film-induced tourism in Asia: A case study of Korean television drama and female viewers’ motivation to visit Korea. *Tourism Culture & Communication*, *7*(3), 207–224. <https://doi.org/10.3727/109830407782212510>
17. Chang, K.-C., Cheng, Y.-S., Kuo, N.-T., & Cheng, Y.-H. (2025). Impacts of tourists’ shopping destination trust on post-visit behaviors: A loss aversion perspective. *International Journal of Hospitality & Tourism Administration*, 1–40. <https://doi.org/10.1080/15256480.2023.2241030>
18. Chang, J., Li, J., & Cheng, S. (2025). Seeing means feeling? The transformation mechanism from visual attention to emotional experience toward linguistic landscape in cultural district. *Journal of Destination Marketing & Management,* 37, 101007–101007. https://doi.org/10.1016/j.jdmm.2025.101007
19. Chen, X., Lee, G., & Joo, D. (2024). Natural landscape performance: Environmental restorativeness and its influence on tourist behavior. *˜the œInternational Journal of Tourism Research (Print)*, *26*(2). <https://doi.org/10.1002/jtr.2636>
20. Chen, Y. C., King, B., & Lee, H.-W. (2018). Experiencing the destination brand: Behavioral intentions of arts festival tourists. *Journal of Destination Marketing & Management*, *10*, 61–67. <https://doi.org/10.1016/j.jdmm.2018.06.004>
21. Cheng, C.-K., & Kuo, H.-Y. (2015). Bonding to a new place never visited: Exploring the relationship between landscape elements and place bonding. *Tourism Management*, *46*(3), 546–560. <https://doi.org/10.1016/j.tourman.2014.08.006>
22. Cheng, Y., Wei, W., & Zhang, L. (2020). Seeing destinations through vlogs: Implications for leveraging customer engagement behavior to increase travel intention. *International Journal of Contemporary Hospitality Management*, *32*(10). <https://doi.org/10.1108/ijchm-04-2020-0319>
23. Cho, K. W., & Frochot, I. (2025). Gone With the Wind? A Longitudinal Analysis of Tourists’ Long-Term Recollections of Holiday Experiences. *Journal of Hospitality & Tourism Research.* https://doi.org/10.1177/10963480251328380
24. Choi, H., & Choi, H. C. (2019). Investigating tourists’ fun-eliciting process toward tourism destination sites: An application of cognitive appraisal theory. *Journal of Travel Research*, *58*(5), 732–744. <https://doi.org/10.1177/0047287518776805>
25. Christou, P., Farmaki, A., & Evangelou, G. (2018). Nurturing nostalgia?: A response from rural tourism stakeholders. *Tourism Management*, *69*(2), 42–51. <https://doi.org/10.1016/j.tourman.2018.05.010>
26. Christou, P., Sharpley, R., & Farmaki, A. (2018). Exploring the emotional dimension of visitors’ satisfaction at cultural events. *Event Management*, *22*(2), 255–269. <https://doi.org/10.3727/152599518x15173355843389>
27. Coronel, M., & Irimiás, A. (2023). “A very special song from Queen to you!” The role of music in destination promotional videos. *Consumer Behavior in Tourism and Hospitality*, *18*(4). <https://doi.org/10.1108/cbth-11-2021-0265>
28. Crespi-Vallbona, M., Noguer-Juncà, E., & Coromina, L. (2023). The destination attachment cycle. The case of academic tourism. *Journal of Tourism and Cultural Change*, *21*(1), 1–18. <https://doi.org/10.1080/14766825.2022.2062247>
29. Dai, Y.-D., Yeh, S.-S., Phuong, L., Wang, T.-S., & Huan, T.-C. (2025). Nostalgic tourists acquiring resilience and emotional solidarity through self-congruity and functional congruity. *Journal of Vacation Marketing, 31(4).* https://doi.org/10.1177/13567667241248964
30. Dalmazi, M. D., Mandolfo, M., Guixeres, J., Raya, M. A., & Lamberti, L. (2024). How immersive technologies impact behavioral responses in destination marketing: the role of physiological arousal, presence, and age. *International Journal of Contemporary Hospitality Management*, *36*(11), 3628–3650. <https://doi.org/10.1108/ijchm-09-2023-1511>
31. Das, A., Banerji, D., Sen, S., & Kondasani, R. K. R. (2025). What Drives Active Loyalty in Tourists? Exploring the Interplay of Tourist Interactions and Experiences, Place Identity, and Customer Perceived Value. *International Journal of Tourism Research, 27(3).* https://doi.org/10.1002/jtr.70069
32. De-Frutos-Arranz, S., & Blasco LópeZ, M.-F. (2022). The state of the art of emotional advertising in tourism: A neuromarketing perspective. *Tourism Review International*, *26*(2). <https://doi.org/10.3727/154427221x16317419620246>
33. Deb, M., & Lomo-David, E. (2021). Determinants of word of mouth intention for a world heritage site: The case of the Sun Temple in India. *Journal of Destination Marketing & Management*, *19*(3), 100533. <https://doi.org/10.1016/j.jdmm.2020.100533>
34. del Bosque, I. R., & Martín, H. S. (2008). Tourist satisfaction a cognitive-affective model. *Annals of Tourism Research*, *35*(2), 551–573. <https://doi.org/10.1016/j.annals.2008.02.006>
35. Deng, Y., Wang, X., & Ma, C. (2024). The Impact of Sports Tourism Motivation on Tourist Loyalty: The Chain Mediation Effect of Experience Quality and Tourist Satisfaction. *International Journal of Tourism Research, 26(5).* https://doi.org/10.1002/jtr.2769
36. Dickinger, A., & Lalicic, L. (2016). An analysis of destination brand personality and emotions: comparison study. *Information Technology & Tourism*, *15*(4), 317–340. <https://doi.org/10.1007/s40558-015-0044-x>
37. Domínguez-Quintero, A. M., González-Rodríguez, M. R., & Roldán, J. L. (2019). The role of authenticity, experience quality, emotions, and satisfaction in a cultural heritage destination. *Journal of Heritage Tourism*, *14*(5-6), 491–505. <https://doi.org/10.1080/1743873x.2018.1554666>
38. Duong, L. H., Kong, Y.-Q., Olya, H., Lee, C.-K., & Girish, V. G. (2025). Echoes of tragedy: How negative social media shapes tourist emotions and avoidance intensions? A multi-methods approach. Tourism Management, 108, 105122. https://doi.org/10.1016/j.tourman.2024.105122
39. Dutta, S., & Dixit, S. (2025a). Examining the impact of media type and perceived Interactivity on Virtual Tour Intentions: empirical evidence using S-O-R framework. Asia Pacific Journal of Tourism Research, 1–20. https://doi.org/10.1080/10941665.2025.2486025
40. Dutta, S., & Dixit, S. (2025b). The psychological influence of perceived destination attributes in 360° virtual tours on tourists’ responses. Tourism Recreation Research, 50(5), 1–14. https://doi.org/10.1080/02508281.2024.2392061
41. Egger, R., & Yu, J. (2025). The impact of real-time hyper-personalisation in AI-generated tourism images. *Journal of Hospitality and Tourism Technology.* https://doi.org/10.1108/jhtt-06-2025-0464
42. Elbaz, A. M., Abou Kamar, M. S., Onjewu, A.-K. E., & Soliman, M. (2023). Evaluating the antecedents of health destination loyalty: The moderating role of destination trust and tourists’ emotions. *International Journal of Hospitality & Tourism Administration*, *24*(1), 1–28. <https://doi.org/10.1080/15256480.2021.1935394>
43. Ene, D., & Schofield, P. (2011). An integrated approach to consumer decision making for budget city breaks: The role of emotion. *International Journal of Tourism Research*, *13*(4), 368–383. <https://doi.org/10.1002/jtr.855>
44. Fan, X., Li, H., & Zheng, S. (2025). Revealing tourists’ appraisal process and consequences of unethical tourism incidents: based on a mixed-method study. *Current Issues in Tourism,* 1–16. https://doi.org/10.1080/13683500.2025.2517791
45. Fan, Y., Wong, I. A., & Lin, Z. (CJ). (2023). How folk music induces destination image: A synthesis between sensory marketing and cognitive balance theory. *Tourism Management Perspectives*, *47*(6), 101123. <https://doi.org/10.1016/j.tmp.2023.101123>
46. Fang, D., Zhao, Z., & Xiong, C. (2024). What leads to an immersive night tourism experience? The relevance of multi-sensory stimuli, emotional involvement, and delight. *Asia Pacific Journal of Tourism Research*, *29*(1), 1–16. <https://doi.org/10.1080/10941665.2024.2308853>
47. Farhat, Z., & Chaney, D. (2021). Introducing destination brand hate: An exploratory study. *Current Issues in Tourism*, *24*(2), 1–17. <https://doi.org/10.1080/13683500.2020.1844160>
48. Filieri, R., Yen, D. A., & Yu, Q. (2021). #ILoveLondon: An exploration of the declaration of love towards a destination on Instagram. *Tourism Management*, *85*(10), 104291. <https://doi.org/10.1016/j.tourman.2021.104291>
49. Filipa, D., Rui, Chim‐Miki, A. F., & Kozak, M. (2025). What Emotions Trigger the Perceived Destination Image and Word‐of‐Mouth Recommendation in World Heritage Sites? *International Journal of Tourism Research, 27(3)*. https://doi.org/10.1002/jtr.70065
50. Folgado-Fernández, J. A., Duarte, P., & Hernández-Mogollón, J. M. (2021). Tourist’s rational and emotional engagement across events: a multi-event integration view. *International Journal of Contemporary Hospitality Management*, *33*(7), 2371–2390. <https://doi.org/10.1108/ijchm-09-2020-1098>
51. Fu, X. (2025). The impact of tourists’ online and offline factors on word of mouth at heritage sites: Imagination, affection, authenticity, and aesthetics. *Tourism Management Perspectives, 59*, 101416. <https://doi.org/10.1016/j.tmp.2025.101416>
52. Ghosh, T., & Sarkar, A. (2016). “To feel a place of heaven”: Examining the role of sensory reference cues and capacity for imagination in destination marketing. *Journal of Travel & Tourism Marketing*, *33*(sup1), 25–37. <https://doi.org/10.1080/10548408.2014.997962>
53. González-Rodríguez, M. R., Domínguez-Quintero, A. M., & Paddison, B. (2020). The direct and indirect influence of experience quality on satisfaction: the importance of emotions. *Current Issues in Tourism*, *23*(22), 2779–2797. <https://doi.org/10.1080/13683500.2019.1668917>
54. Goossens, C. (2000). Tourism information and pleasure motivation. *Annals of Tourism Research*, *27*(2), 301–321. <https://doi.org/10.1016/s0160-7383(99)00067-5>
55. Hadinejad, A., Gardiner, S., Kralj, A., Scott, N., & Moyle, B. D. (2022). Cognition, metacognition and attitude to tourism destinations: The impact of emotional arousal and source credibility. *Journal of Hospitality and Tourism Management*, *51*(6), 502–511. <https://doi.org/10.1016/j.jhtm.2022.05.005>
56. Hamid, A. H. A., Mohamad, M. R., & Suki, N. M. (2021). Tourists’ revisit intention to UNESCO world heritage sites in a developing nation: Investigating the mediating role of place dependence. *Journal of Vacation Marketing*, *27*(1), 135676672096973. <https://doi.org/10.1177/1356766720969739>
57. He, M., Tu, J., Li, Y., Fang, J., & Zeng, X. (2024). Tourist preference for tourism product brand biographies. *Annals of Tourism Research*, *107*, 103773. <https://doi.org/10.1016/j.annals.2024.103773>
58. Heredia-Carroza, J., Peña-Vinces, J., Palma, L., & Aguado, L. F. (2025). The impact of anticipated feelings and artist fame on customers’ decisions to attend live flamenco shows. *Journal of Destination Marketing & Management, 36,* 100994. https://doi.org/10.1016/j.jdmm.2025.100994
59. Hosany, S. (2012). Appraisal determinants of tourist emotional responses. *Journal of Travel Research*, *51*(3), 303–314. <https://doi.org/10.1177/0047287511410320>
60. Hosany, S., & Gilbert, D. (2010). Measuring tourists’ emotional experiences toward hedonic holiday destinations. *Journal of Travel Research*, *49*(4), 513–526. <https://doi.org/10.1177/0047287509349267>
61. Hosseini, S., Cortes-Macías, R., & Almeida-García, F. (2024). Extending the memorable tourism experience construct: An investigation of tourists’ memorable dark experiences. *Journal of Vacation Marketing*, *30*(1), 135676672211130. <https://doi.org/10.1177/13567667221113078>
62. Hosseini, S., María Jesús Carrasco-Santos, Erose Sthapit, & Pere Mercadé-Melé. (2025). Reimagining travel memories: the role of artificial intelligence in shaping tourist experiences. *Current Issues in Tourism,* 1–24. https://doi.org/10.1080/13683500.2025.2576659
63. Hsu, F.-C., Liu, J., & Lin, H. (2022). Affective components of gastronomy tourism: Measurement scale development and validation. *International Journal of Contemporary Hospitality Management*, *34*(9), 3278–3299. <https://doi.org/10.1108/ijchm-09-2021-1112>
64. Hu, M., Zhang, Y., Zhang, H., Lu, Y., Zuo, L., Zhuang, M., Liu, W., Zhang, J., & Zhang, H. (2020). How do Chinese tourists perceive tranquillity during the tour? *Tourism Management Perspectives*, *34*, 100666. <https://doi.org/10.1016/j.tmp.2020.100666>
65. Huang, T., & Zhang, Y. (2024). “Roots” tourists’ personal heritage experience: An extended cognitive-affective-conative model. *Journal of Hospitality and Tourism Management, 61,* 212–223. https://doi.org/10.1016/j.jhtm.2024.10.009
66. Huang, Y.-C., Backman, S. J., Backman, K. F., & Moore, D. (2013). Exploring user acceptance of 3D virtual worlds in travel and tourism marketing. *Tourism Management*, *36*(1), 490–501. <https://doi.org/10.1016/j.tourman.2012.09.009>
67. Huseynov, K., Costa Pinto, D., Maurer Herter, M., & Rita, P. (2020). Rethinking emotions and destination experience: An extended model of goal-directed behavior. *Journal of Hospitality & Tourism Research*, *44*(7), 1153–1177. <https://doi.org/10.1177/1096348020936334>
68. Ito, E., Kono, S., & Gui, J. (2023). Psychological consequences of tourism ideal affect. *Current Issues in Tourism*, *23*(6), 1–12. <https://doi.org/10.1080/13683500.2021.2023479>
69. Japutra, A., & Keni, K. (2020). Signal, need fulfilment and tourists’ intention to revisit. *Anatolia*, *31*(2), 1–15. <https://doi.org/10.1080/13032917.2020.1806889>
70. Jaya, I., & Jaw, C. (2024). Innovativeness or involvement: how virtual reality influences nostalgic emotion and imagery in travel intention. *Journal of Hospitality & Tourism Research*, *48*(6). <https://doi.org/10.1177/10963480231194692>
71. Jiang, G.-X., Li, Y.-Q., Ruan, W.-Q., & Zhang, S.-N. (2024). Relieving tourist anxiety during the COVID-19 epidemic: a dual perspective of the government and the tourist destination. *Current Issues in Tourism, 27(7)*, 1–16. https://doi.org/10.1080/13683500.2023.2198118
72. Jiang, J., & Yan, B. (2022). From soundscape participation to tourist loyalty in nature-based tourism: The moderating role of soundscape emotion and the mediating role of soundscape satisfaction. *Journal of Destination Marketing & Management*, *26*(2), 100730. <https://doi.org/10.1016/j.jdmm.2022.100730>
73. Jiang, L., & Yu, L. (2020). Consumption of a literary tourism place: a perspective of embodiment. *Tourism Geographies*, *22*(1), 127–150. <https://doi.org/10.1080/14616688.2019.1586985>
74. Jiang, M., & Garrod, B. (2025). Stirring the emotions: modelling the role of emotions in developing satisfaction and loyalty in tea tourism experiences. *Current Issues in Tourism,* 1–17. https://doi.org/10.1080/13683500.2025.2478285
75. Jiang, Y., Ramkissoon, H., & Mavondo, F. (2015). Destination marketing and visitor experiences: The development of a conceptual framework. *Journal of Hospitality Marketing & Management*, *25*(6), 653–675. <https://doi.org/10.1080/19368623.2016.1087358>
76. Joo, D., Cho, H., & Woosnam, K. M. (2019). Exploring tourists’ perceptions of tourism impacts. *Tourism Management Perspectives*, *31*, 231–235. <https://doi.org/10.1016/j.tmp.2019.05.008>
77. Karagöz, D., & Uysal, M. (2022). Tourists’ need for uniqueness as a representation of differentiated identity. *Journal of Travel Research*, *61*(1), 004728752097280. <https://doi.org/10.1177/0047287520972804>
78. Kastenholz, E., Marques, C. P., & Carneiro, M. J. (2020). Place attachment through sensory-rich, emotion-generating place experiences in rural tourism. *Journal of Destination Marketing & Management*, *17*(2), 100455. <https://doi.org/10.1016/j.jdmm.2020.100455>
79. Keskin, E., Aktaş, F., Yayla, Ö., & Dedeoğlu, B. B. (2024). The Importance of nostalgic emotions and memorable tourism experience in the cultural experience. *Journal of Quality Assurance in Hospitality & Tourism*, *25*(1), 1–21. <https://doi.org/10.1080/1528008x.2022.2097976>
80. Ketter, E. (2018). It’s all about you: destination marketing campaigns in the experience economy era. *Tourism Review*, *73*(3), 331–343. <https://doi.org/10.1108/tr-03-2017-0053>
81. Kil, N., Holland, S. M., Stein, T. V., & Ko, Y. J. (2012). Place attachment as a mediator of the relationship between nature-based recreation benefits and future visit intentions. *Journal of Sustainable Tourism*, *20*(4), 603–626. <https://doi.org/10.1080/09669582.2011.610508>
82. Kim, E., Kim, S., & Jeong, Y. (2024). The 18th FINA world masters championships and destination loyalty. *Asia Pacific Journal of Marketing and Logistics, 36(3),* 622–640. https://doi.org/10.1108/apjml-04-2023-0294
83. Kim, H., & Stepchenkova, S. (2017). Understanding destination personality through visitors’ experience: A cross-cultural perspective. *Journal of Destination Marketing & Management*, *6*(4), 416–425. <https://doi.org/10.1016/j.jdmm.2016.06.010>
84. Kim, J.-H. (2022). Destination Attributes Affecting Negative Memory: Scale Development and Validation. *Journal of Travel Research*, *61*(4), 004728752097772. <https://doi.org/10.1177/0047287520977725>
85. Kim, J.-H., Guo, J., & Wang, Y. (2022). Tourists’ negative emotions: antecedents and consequences. *Current Issues in Tourism*, *25*(1), 1–19. <https://doi.org/10.1080/13683500.2021.1935793>
86. Kim, J.-H., & Youn, H. (2017). How to design and deliver stories about tourism destinations. *Journal of Travel Research*, *56*(6), 808–820. <https://doi.org/10.1177/0047287516666720>
87. Kim, S.-S., Shin, W., & Kim, H.-W. (2025). Unravelling long-stay tourist experiences and satisfaction: text mining and deep learning approaches. *Current Issues in Tourism, 28(3),* 1–19. https://doi.org/10.1080/13683500.2024.2327840
88. Kucukergin, K. G., Kucukergin, F. N., & Dedeoglu, B. B. (2020). An overview of the destination physical servicescape with SOR paradigm: the importance of prestige sensitivity. *Asia Pacific Journal of Tourism Research*, *25*(5), 473–488. <https://doi.org/10.1080/10941665.2020.1737160>
89. Le, D., Scott, N., & Wang, Y. (2021). Impact of prior knowledge and psychological distance on tourist imagination of a promoted tourism event. *Journal of Hospitality and Tourism Management*, *49*(3), 101–111. <https://doi.org/10.1016/j.jhtm.2021.09.001>
90. Lee, J. (Jiyeon), Kyle, G., & Scott, D. (2012). The mediating effect of place attachment on the relationship between festival satisfaction and loyalty to the festival hosting destination. *Journal of Travel Research*, *51*(6), 754–767. <https://doi.org/10.1177/0047287512437859>
91. Lee, K.-H., & Hyun, S. S. (2016). The effects of perceived destination ability and destination brand love on tourists’ loyalty to post-disaster tourism destinations: The case of Korean tourists to Japan. *Journal of Travel & Tourism Marketing*, *33*(5), 613–627. <https://doi.org/10.1080/10548408.2016.1167349>
92. Lee, S., Kim, T., & Young Hoon Kim. (2025). Heritage tourism and allegiance development: A study of the Korean demilitarized zone. *Journal of Vacation Marketing*. <https://doi.org/10.1177/13567667241249178>
93. Letheren, K., Martin, B. A. S., & Jin, H. S. (2017). Effects of personification and anthropomorphic tendency on destination attitude and travel intentions. *Tourism Management*, *62*, 65–75. <https://doi.org/10.1016/j.tourman.2017.03.020>
94. Li, G., Zhang, C., Xu, D., & Wang, L. (2024). How emotions catalyse learning through study tours: Evidence from Panda Ambassador. *Journal of Hospitality and Tourism Management*, *60*(5), 322–333. <https://doi.org/10.1016/j.jhtm.2024.08.006>
95. Li, S. (2019). Emotional appeals in tourism TV commercials: A psycho-physiological study. *Journal of Hospitality & Tourism Research*, *43*(6), 783–806. <https://doi.org/10.1177/1096348019828440>
96. Li, L., Liu, M., Zhang, Y., & Lai, X. (2025). How can use and gratification attributes evoke on-site travel intention in digital museum tourism? A serial mediation model. *Journal of Hospitality and Tourism Technology,* 1–23. https://doi.org/10.1108/jhtt-08-2024-0534
97. Li, S., Walters, G., Packer, J., & Scott, N. (2018). A comparative analysis of self-report and psychophysiological measures of emotion in the context of tourism advertising. *Journal of Travel Research*, *57*(8), 1078–1092. <https://doi.org/10.1177/0047287517733555>
98. Li, S., Walters, G., Packer, J., & Scott, N. (2022). Using facial electromyography to test the peak–end rule in tourism advertising. *Journal of Hospitality & Tourism Research*, *46*(3), 109634801989004. <https://doi.org/10.1177/1096348019890047>
99. Li, S., Zheng, D., & Zhan, J. (2024). Emotional dynamics of virtual reality tourism experience: An application of day reconstruction and physiological methods. *International Journal of Tourism Research*, *26*(4). <https://doi.org/10.1002/jtr.2707>
100. Li, X., Kong, W. H., & Yang, F. X. (2021). Authentic food experiences bring us back to the past: an investigation of a local food night market. *Journal of Travel & Tourism Marketing*, *38*(3), 233–246. <https://doi.org/10.1080/10548408.2021.1902910>
101. Li, X., Yuan, Y., & Zhang, J. (2024). The influence of tourists’ emotional experiences on destination loyalty from the perspective of community economy. *International Journal of Tourism Research, 26(1)*. https://doi.org/10.1002/jtr.2630
102. Li, Z., & Zhang, J. (2023). How to improve destination brand identification and loyalty using short-form videos? The role of emotional experience and self-congruity. *Journal of Destination Marketing & Management*, *30*(1), 100825. <https://doi.org/10.1016/j.jdmm.2023.100825>
103. Liu, J., Zhao, J., Wei, W., & Yang, T. (2025). Captivating the crowd: unraveling sentiments in tourism short videos for effective destination marketing on social media. *Current Issues in Tourism,* 1–28. https://doi.org/10.1080/13683500.2025.2462167
104. Liu, L., Cui, T., Wu, J., Cao, R., & Ye, Y. (2020). Encouraging tourist citizenship behavior through resource uniqueness and service quality: The mediating role of emotions. *Journal of Vacation Marketing*, *27*(1), 45–60. <https://doi.org/10.1177/1356766720952101>
105. Loureiro, S. M. C. (2015). The role of website quality on PAD, attitude and intentions to visit and recommend island destination. *International Journal of Tourism Research*, *17*(6), 545–554. <https://doi.org/10.1002/jtr.2022>
106. Lv, X., & Wu, A. (2021). The role of extraordinary sensory experiences in shaping destination brand love: An empirical study. *Journal of Travel & Tourism Marketing*, *38*(2), 179–193. <https://doi.org/10.1080/10548408.2021.1889447>
107. M. Bahadır Kalıpçı. (2024). Emotions or numbers in tourism advertisements? A comprehensive analysis of two neighboring destinations in the pre- and post-COVID-19 period. *International Journal of Tourism Cities*, *10*(3), 817–841. <https://doi.org/10.1108/ijtc-08-2023-0167>
108. Ma, J., Gao, J., Scott, N., & Ding, P. (2013). Customer delight from theme park experiences. *Annals of Tourism Research*, *42*(1), 359–381. <https://doi.org/10.1016/j.annals.2013.02.018>
109. Ma, J., Li, F. (Sam), & Shang, Y. (2022). Tourist scams, moral emotions and behaviors: impacts on moral emotions, dissatisfaction, revisit intention and negative word of mouth. *Tourism Review*, *77*(2). <https://doi.org/10.1108/tr-03-2022-0115>
110. Manner-Baldeon, F., & Li, M. (2024). Traveling to the danger zone: Emotional dynamics and hermeneutics. *Journal of Hospitality & Tourism Research*, *48*(3). <https://doi.org/10.1177/10963480231220267>
111. McClinchey, K. A. (2015). Travel books, place experience and the “Je ne sais quoi” of Paris. *Tourism Geographies*, *17*(5), 701–718. <https://doi.org/10.1080/14616688.2015.1066843>
112. McMullan, R., & O’Neill, M. (2010). Towards a valid and reliable measure of visitor satisfaction. *Journal of Vacation Marketing*, *16*(1), 29–44. <https://doi.org/10.1177/1356766709356141>
113. Mehra, P. (2023). Unexpected surprise: Emotion analysis and aspect based sentiment analysis (ABSA) of user generated comments to study behavioral intentions of tourists. *Tourism Management Perspectives*, *45*(7), 101063. <https://doi.org/10.1016/j.tmp.2022.101063>
114. Meng, B., & Luo, D. (2024). Family tourists’ emotional responses at world heritage sites (WHSs): The effects of existential authenticity and interpersonal interaction. *Tourism Review*, *79*(3). <https://doi.org/10.1108/tr-01-2023-0053>
115. Michalkó, G., Irimiás, A., & Timothy, D. J. (2015). Disappointment in tourism: Perspectives on tourism destination management. *Tourism Management Perspectives*, *16*, 85–91. <https://doi.org/10.1016/j.tmp.2015.07.007>
116. Min, Z., Jie, Z., Xiao, X., Mengyuan, Q., Youhai, L., Hui, Z., Tz-Hsuan, T., Lin, Z., & Meng, H. (2020). How destination music affects tourists’ behaviors: travel with music in Lijiang, China. *Asia Pacific Journal of Tourism Research*, *25*(2), 131–144. <https://doi.org/10.1080/10941665.2019.1683046>
117. Mionel, V. (2020). (Not so) Dark tourism: The Merry Cemetery in Săpânţa (Romania) – An expression of folk culture. *Tourism Management Perspectives*, *34*(1), 100656. <https://doi.org/10.1016/j.tmp.2020.100656>
118. Mirzamurodova Kizi, A. A., Zielinski, S., Milanes, C. B., & Ahn, Y. (2025). Tourist Destination Image Projected by YouTube Travel Videos: The Case of Seoul, Republic of Korea. *International Journal of Tourism Research, 27(3)*. https://doi.org/10.1002/jtr.70049
119. Morrison, A. M., Bag, S., & Mandal, K. (2024). Virtual reality’s impact on destination visit intentions and the moderating role of amateur photography. *Tourism Review*, *79*(2). <https://doi.org/10.1108/tr-12-2022-0621>
120. Mura, P. (2010). “Scary … but I like it!” Young tourists’ perceptions of fear on holiday. *Journal of Tourism and Cultural Change*, *8*(1-2), 30–49. <https://doi.org/10.1080/14766825.2010.482209>
121. Murphy, L., Benckendorff, P., & Moscardo, G. (2007). Linking travel motivation, tourist self-image and destination brand personality. *Journal of Travel & Tourism Marketing*, *22*(2), 45–59. <https://doi.org/10.1300/j073v22n02_04>
122. Nadeau, J., Wardley, L. J., & Rajabi, E. (2022). Tourism destination image resiliency during a pandemic as portrayed through emotions on Twitter. *Tourism and Hospitality Research*, *22*(1), 146735842110383. <https://doi.org/10.1177/14673584211038317>
123. Nhat, A., Thi, P., & Thanh Dat Le. (2024). Unlocking the precursors of destination brand love: the roles of self-congruence and destination brand immersion. *Journal of Hospitality and Tourism Insights*, *7*(2). <https://doi.org/10.1108/jhti-01-2024-0021>
124. Niusha Talebzadeh, Ilkay Yorganci, Nosrati, S., & Kilic, C. (2024). Residents’ emotional and behavioral responses to visiting Maras (Varosha) as a dark tourism spot. *Journal of Heritage Tourism*, *19*(3), 1–24. <https://doi.org/10.1080/1743873x.2024.2302535>
125. Otay Demir, F., Yavuz Görkem, Ş., & Rafferty, G. (2022). An inquiry on the potential of computational literary techniques towards successful destination branding and literary tourism. *Current Issues in Tourism*, *25*(5), 764–778. <https://doi.org/10.1080/13683500.2021.1887100>
126. Pandža Bajs, I. (2015). Tourist perceived value, relationship to satisfaction, and behavioral intentions. *Journal of Travel Research*, *54*(1), 122–134. <https://doi.org/10.1177/0047287513513158>
127. Patwardhan, V., Ribeiro, M. A., Woosnam, K. M., Payini, V., & Mallya, J. (2020). Visitors’ loyalty to religious tourism destinations: Considering place attachment, emotional experience and religious affiliation. *Tourism Management Perspectives*, *36*(3), 100737. <https://doi.org/10.1016/j.tmp.2020.100737>
128. Peng, J., Yang, X., Fu, S., & Huan, T.-C. (T. C. ). (2023). Exploring the influence of tourists’ happiness on revisit intention in the context of Traditional Chinese Medicine cultural tourism. *Tourism Management*, *94*, 104647. <https://doi.org/10.1016/j.tourman.2022.104647>
129. Pimentel Biscaia, M. S., & Marques, L. (2022). Dystopian dark tourism: affective experiences in Dismaland. *Tourism Geographies*, *24*(3), 1–20. <https://doi.org/10.1080/14616688.2020.1795710>
130. Prayag, G., Hosany, S., Muskat, B., & Del Chiappa, G. (2017). Understanding the relationships between tourists’ emotional experiences, perceived overall image, satisfaction, and intention to recommend. *Journal of Travel Research*, *56*(1), 41–54. <https://doi.org/10.1177/0047287515620567>
131. Prayag, G., Hosany, S., & Odeh, K. (2013). The role of tourists’ emotional experiences and satisfaction in understanding behavioral intentions. *Journal of Destination Marketing & Management*, *2*(2), 118–127. <https://doi.org/10.1016/j.jdmm.2013.05.001>
132. Prayag, G., Pung, J. M., Lee, C., & Del Chiappa, G. (2022). The self-concept and psychological antecedents of intention to recommend a heritage site: The moderating effects of visitor type. *Tourism Management Perspectives*, *42*(2), 100962. <https://doi.org/10.1016/j.tmp.2022.100962>
133. Qian, L., Guo, J., Hanqin Qiu Zhang, Zheng, C., & Ren, L. (2023). Exploring destination image of dark tourism via analyzing user generated photos: A deep learning approach. *Tourism Management Perspectives*, *48*(4), 101147–101147. <https://doi.org/10.1016/j.tmp.2023.101147>
134. Qiu, M., Zhang, J., & Zheng, C. (2018). Exploring tourists’ soundscape emotion and its impact on sustainable tourism development. *Asia Pacific Journal of Tourism Research*, *23*(9), 862–879. <https://doi.org/10.1080/10941665.2018.1494614>
135. Qiu, Q., & Zuo, Y. (2023). “Intangible cultural heritage” label in destination marketing toolkits: Does it work and how?. *Journal of Hospitality and Tourism Management*, *56*, 272–283. <https://doi.org/10.1016/j.jhtm.2023.06.025>
136. Qu, Y., & Dong, Y. (2025). The Embodied Formation of Destination Attachment. *Journal of Travel Research.* https://doi.org/10.1177/00472875251332947
137. Rahmani, K., Gnoth, J., & Mather, D. (2019). A Psycholinguistic View of Tourists’ Emotional Experiences. *Journal of Travel Research*, *58*(2), 192–206. <https://doi.org/10.1177/0047287517753072>
138. Ramírez-Gutiérrez, D., Fernández-Betancort, H., & Santana-Talavera, A. (2018). Talking to others: Analysing tourists’ communications on cultural heritage experiences. *International Journal of Heritage Studies*, *24*(6), 634–648. <https://doi.org/10.1080/13527258.2017.1413674>
139. Rezaei, N., Huang, W. (Sabrina), & Hung, K. (2023a). Antecedents and outcome of tourists’ positive achievement emotions in visiting risky destinations: The case of Middle East. *International Journal of Tourism Research*. <https://doi.org/10.1002/jtr.2575>
140. Rezaei, N., Huang, W., & Hung, K. (2023b). Tourist’s achievement emotions and memorable experience in visiting the Middle East. *Tourism Management Perspectives*, *47*(8), 101129–101129. <https://doi.org/10.1016/j.tmp.2023.101129>
141. Riena Thongtammachat, Chen, T., & Osmond, A. (2023). Fun or Fear? On-Site Versus On-Screen Experiences in Paranormal Tourism: A Case Study of Hashima Island, Japan. *Tourism Review International*, *27*(3), 217–234. <https://doi.org/10.3727/154427223x16819417821705>
142. Rodrigues, Á., Pereira, R., & Rodrigues, A. I. (2020). Exploring the potential of nightscape photography for tourism: Preliminary insights. *Tourism Analysis*, *25*. <https://doi.org/10.3727/108354220x15758301241738>
143. Rotem Mashkov, & Noam Shoval. (2025). Measurement of Visitors’ Subjective and Objective Emotional Response to Congestion in Public Spaces. *Journal of Travel Research, 64(6)*. https://doi.org/10.1177/00472875241249385
144. Royo-Vela, M. (2009). Rural-cultural excursion conceptualization: A local tourism marketing management model based on tourist destination image measurement. *Tourism Management*, *30*(3), 419–428. <https://doi.org/10.1016/j.tourman.2008.07.013>
145. Ruan, W.-Q., Tang, Y.-T., Zhang, S.-N., & Wang, M.-Y. (2025). “Poetic” marketing: How language makes destinations beloved. *Journal of Hospitality and Tourism Management, 65*, 101340–101340. https://doi.org/10.1016/j.jhtm.2025.101340
146. Sajad Ferdowsi, & Hosseinzadeh, H. (2025). Digital content marketing in geotourism: bridging online engagement and destination choice. *Current Issues in Tourism*, 1–20. https://doi.org/10.1080/13683500.2025.2571868
147. S. Mostafa Rasoolimanesh, & Lu, S. (2024). Enhancing emotional responses of tourists in cultural heritage tourism: the case of Pingyao, China. *Journal of Heritage Tourism*, *19*(1), 1–20. <https://doi.org/10.1080/1743873x.2023.2254420>
148. Šagovnović, I., & Kovačić, S. (2021). Influence of tourists’ sociodemographic characteristics on their perception of destination personality and emotional experience of a city break destination. *International Journal of Tourism Cities*, *7*(1), 200–223. <https://doi.org/10.1108/ijtc-05-2020-0105>
149. Şahin, İ., & Güzel, F. Ö. (2020). Do experiential destination attributes create emotional arousal and memory? : A comparative research approach. *Journal of Hospitality Marketing & Management*, *29*(8), 1–31. <https://doi.org/10.1080/19368623.2020.1746214>
150. Saidi, H. (2013). From Québec to Carthage or how to be there when you are here. *Anatolia*, *24*(3), 381–394. <https://doi.org/10.1080/13032917.2013.770768>
151. Sann, R., Pakkapol Luecha, & Rawisara Rueangchaithanakun. (2024). The effects of virtual reality travel on satisfaction and visiting intention utilizing an extended stimulus-organism-response theory: perspectives from Thai tourists. *Journal of Hospitality and Tourism Insights*, *7*(3). <https://doi.org/10.1108/jhti-05-2023-0321>
152. Sarker, T., Rabbanee, F. K., Aishath Shakeela, Jebarajakirthy, C., Sarwar, A., & Amin, R. (2023). Understanding the drivers of destination equity in the post-pandemic era: the case of Australia. *Asia Pacific Journal of Tourism Research*, *28*(5), 433–448. <https://doi.org/10.1080/10941665.2023.2245501>
153. Schoner-Schatz, L., Hofmann, V., & Stokburger-Sauer, N. E. (2021). Destination’s social media communication and emotions: An investigation of visit intentions, word-of-mouth and travelers’ facially expressed emotions. *Journal of Destination Marketing & Management*, *22*(1), 100661. <https://doi.org/10.1016/j.jdmm.2021.100661>
154. Servidio, R. (2015). Images, affective evaluation and personality traits in tourist behaviour: An exploratory study with Italian postcards. *Tourism Management Perspectives*, *16*, 237–246. <https://doi.org/10.1016/j.tmp.2015.08.003>
155. Sharma, P., & Nayak, J. K. (2019). Do tourists’ emotional experiences influence images and intentions in yoga tourism? *Tourism Review*, *74*(3), 646–665. <https://doi.org/10.1108/tr-05-2018-0060>
156. Shi, Y., Bettache, K., Zhang, N., & Xue, L. (2021). Constructing nostalgia in tourism: A comparison analysis of genuine and artificial approaches. *Journal of Destination Marketing & Management*, *19*(1), 100488. <https://doi.org/10.1016/j.jdmm.2020.100488>
157. Silkes, C. A., Cai, L. A., & Lehto, X. Y. (2013). Marketing to the culinary tourist. *Journal of Travel & Tourism Marketing*, *30*(4), 335–349. <https://doi.org/10.1080/10548408.2013.784151>
158. Silva, R., & Correia, A. (2017). Places and tourists: ties that reinforce behavioural intentions. *Anatolia*, *28*(1), 14–30. <https://doi.org/10.1080/13032917.2016.1240093>
159. Sinha, N., Kapoor, G., & Kataria, N. (2025). Customer Engagement in Virtual Tourism: Embracing Immersive Experience of Culture and Heritage. *International Journal of Tourism Research, 27(4)*. https://doi.org/10.1002/jtr.70094
160. Song, H., & Lu, S. (2024). The effect of virtual tourism experience on tourist responses: the lens from cognitive appraisal theory. *Asia Pacific Journal of Tourism Research*, *27*(9), 1–15. <https://doi.org/10.1080/10941665.2024.2351126>
161. Steriopoulos, E., Khoo, C., Wong, H. Y., Hall, J., & Steel, M. (2024). Heritage tourism brand experiences: The influence of emotions and emotional engagement. *Journal of Vacation Marketing*, *30*(2), 135676672311529. <https://doi.org/10.1177/13567667231152930>
162. Sthapit, E. (2019). Memories of gastronomic experiences, savoured positive emotions and savouring processes. *Scandinavian Journal of Hospitality and Tourism*, *19*(2), 115–139. <https://doi.org/10.1080/15022250.2017.1402702>
163. Su, L., Cheng, J., & Swanson, S. R. (2020). The impact of tourism activity type on emotion and storytelling: The moderating roles of travel companion presence and relative ability. *Tourism Management*, *81*(2), 104138. <https://doi.org/10.1016/j.tourman.2020.104138>
164. Su, L., Swanson, S. R., & Chen, X. (2018). The relationship of perceived service quality with revisitation and subjective well-being of Chinese tourists: The role of emotions and destination identification. *Tourism Review International*, *22*(2), 131–142. <https://doi.org/10.3727/154427218x15319286372289>
165. Syed Ahamed Suban. (2024). Spa tourism: understanding the relationship of tourist`s emotional experience, destination image, satisfaction, and intention to recommend using an integrated model. *International Journal of Spa and Wellness*, *7*(3), 1–22. <https://doi.org/10.1080/24721735.2024.2314943>
166. Taheri, B. (2016). Emotional connection, materialism, and religiosity: An Islamic tourism experience. *Journal of Travel & Tourism Marketing*, *33*(7), 1011–1027. <https://doi.org/10.1080/10548408.2015.1078761>
167. Takata, K., & Hallmann, K. (2025). Experiencing Authenticity at Heritage Sporting Events. *Event Management, 29(4).* https://doi.org/10.3727/152599525x17367484906417
168. Tercia, C., Teichert, T., Sirad, D. A., & Murniadi, K. (2022). Storytelling in the communication of dark tourism. *Consumer Behavior in Tourism and Hospitality*. <https://doi.org/10.1108/cbth-06-2021-0152>
169. Thanh, T., & Dung, T. (2025). The role of nostalgic emotion in shaping destination image and behavioral intentions – An empirical study. *Journal of Hospitality and Tourism Insights*. <https://doi.org/10.1108/jhti-05-2024-0463>
170. Tian, F., Fu, S., Yang, Y., Wang, Z., & Liu, D. (2024). Understanding nostalgia in historical and cultural districts: What can we learn from documentaries? *Tourism Management Perspectives*, *54*(3), 101302. <https://doi.org/10.1016/j.tmp.2024.101302>
171. Tran-tuan-Hung, Schneider, I. E., & Gartner, W. C. (2006). Image of Vietnam held by US tourists: Initial inquiry. *Asia Pacific Journal of Tourism Research*, *11*(2), 147–159. <https://doi.org/10.1080/10941660600727509>
172. Tucker, H., & Shelton, E. J. (2018). Tourism, mood and affect: Narratives of loss and hope. *Annals of Tourism Research*, *70*(3), 66–75. <https://doi.org/10.1016/j.annals.2018.03.001>
173. Vajpai, G. N., Porya, A., & Chowdhary, N. (2025). Strategic touchpoint mapping: advancing destination marketing and management in nature-based tourism. *Tourism Recreation Research,* 1–17. <https://doi.org/10.1080/02508281.2025.2577471>
174. Veenus, T., Azizah, O., Mishra, A., & Saad, M. (2025). Significance of Destination Fascination in Destination Marketing Through Movies. *Tourism Analysis.* https://doi.org/10.3727/108354225x17418823599463
175. Volkan Genç, Anil Bilgihan, Seray Gülertekın Genc, & Fevzi Okumuş. (2023). Seeing history come to life with augmented reality: the museum experience of generation Z in Göbeklitepe. *Journal of Tourism and Cultural Change*, *21*(6), 657–676. <https://doi.org/10.1080/14766825.2023.2213679>
176. Walters, G., Sparks, B., & Herington, C. (2012). The impact of consumption vision and emotion on the tourism consumer’s decision behavior. *Journal of Hospitality & Tourism Research*, *36*(3), 366–389. <https://doi.org/10.1177/1096348010390815>
177. Wang, E., Shen, C., Zheng, J., Wu, D., & Cao, N. (2021). The antecedents and consequences of awe in dark tourism. *Current Issues in Tourism*, *24*(8), 1–15. <https://doi.org/10.1080/13683500.2020.1782857>
178. Wang, X., Han, G., Wang, Y., & Zheng, J. (2024). More than words: Do personality, emotion, and lifestyle matter on the wine tourism? *International Journal of Tourism Research*, *26*(4). <https://doi.org/10.1002/jtr.2667>
179. Wang, H., Liang, Y., Ti, Y., & Lee, T. J. (2025). Cognitive, affective, and behavioral mechanisms in medical tourism from a dual-theory perspective. *Current Issues in Tourism,* 1–25. https://doi.org/10.1080/13683500.2025.2569836
180. Wang, X., Oh, Y., Park, S., Hong, S., & Lai, P. (2024). Factors influencing travelers’ intention by environmental changes of destination: Cross‐country evidence from Far‐East Asia. *International Journal of Tourism Research*, *26*(4). <https://doi.org/10.1002/jtr.2705>
181. Wang, Y., Xiong, M., Miao, H., Wang, Y., Yang, X., & Zhou, J. (2025). Impact of perceived rural destinations restorativeness on revisit intentions: The neglected post-travel negative emotions. *Journal of Hospitality and Tourism Management, 62,* 219–231. https://doi.org/10.1016/j.jhtm.2025.01.014
182. Wang, J., Ding, X., & Wang, K. (2024). Brand gestalt cognition and tourists’destination decision making: a study of international island destinations. *Current Issues in Tourism, 27(18)*, 1–17. <https://doi.org/10.1080/13683500.2023.2245108>
183. Wang, J., Leung, X. Y., & Song, X. (2025). Dual pathways of dark tourism’s impact on tourists’ happiness: The mediating role of mixed emotions. *Journal of Hospitality and Tourism Management, 63*, 247–258. https://doi.org/10.1016/j.jhtm.2025.05.004
184. Wu, X., & Zhao, Y. (2023). An integrated model of five sense perceptions and memorable tourism experiences: Mediating effects of positive emotion. *Asia Pacific Journal of Tourism Research*, *28*(8), 860–876. <https://doi.org/10.1080/10941665.2023.2276473>
185. Xu, D., Murphy, L., & Chen, T. (2022). Tourist scams: cues and processes in decision-making. *Asia Pacific Journal of Tourism Research*, *27*(12), 1247–1263. <https://doi.org/10.1080/10941665.2023.2174037>
186. Xu, F., Zhan, C., Li, J., & Li, S. (2024). Tourists’ approach or avoidance reaction to crowding in wellness tourism. *Journal of Travel & Tourism Marketing, 41(6),* 880–900. https://doi.org/10.1080/10548408.2024.2349814
187. Xu, J. (Bill), Yan, L., & Mak, C. K. Y. (2021). Service encounter failure, negative destination emotion and behavioral intention: An experimental study of taxi service. *Tourism Management Perspectives*, *40*, 100886. <https://doi.org/10.1016/j.tmp.2021.100886>
188. Yamagishi, K., Danzel Canayong, Domingo, M., Kim Nieva Maneja, Montolo, A., & Arabelle Siton. (2024). User-generated content on Gen Z tourist visit intention: a stimulus-organism-response approach. *Journal of Hospitality and Tourism Insights*, *7*(1). <https://doi.org/10.1108/jhti-02-2023-0091>
189. Yan, B., Zhang, J., Jiang, J., Chen, K., & Li, L. (2025). Calligraphic landscapes and environments: perceived stimuli, emotion and commitment in heritage tourism. *Asia Pacific Journal of Tourism Research,* 1–19. https://doi.org/10.1080/10941665.2025.2523337
190. Yan, B.-J., Zhang, J., Zhang, H.-L., Lu, S.-J., & Guo, Y.-R. (2016). Investigating the motivation–experience relationship in a dark tourism space: A case study of the Beichuan earthquake relics, China. *Tourism Management*, *53*(53), 108–121. <https://doi.org/10.1016/j.tourman.2015.09.014>
191. Yan, N., & Halpenny, E. A. (2022). Tourists’ savoring of positive emotions and place attachment formation: a conceptual paper. *Tourism Geographies*, *24*, 1–21. <https://doi.org/10.1080/14616688.2019.1647454>
192. Yang, Y., Ruan, Q., Huang, S. (Sam), Lan, T., & Wang, Y. (2021). Impact of the COVID-19 outbreak on tourists’ real-time on-site emotional experience in reopened tourism destinations. *Journal of Hospitality and Tourism Management*, *48*(2), 390–394. <https://doi.org/10.1016/j.jhtm.2021.07.014>
193. Yao, Y., Xingyang Lv, Ren, L., & Wu, A. (2025). Rhythmic tour: Emotional fluctuation and tourist well-being. *Annals of Tourism Research, 114,* 104002–104002. https://doi.org/10.1016/j.annals.2025.104002
194. Yin, J., Cheng, Y., & Ni, Y. (2024). Staging a comeback? The influencing mechanism of tourist crowding perception on adaptive behavior. *Tourism Management, 100,* 104827–104827. https://doi.org/10.1016/j.tourman.2023.104827
195. Yin, J., Ji, Y., & Ni, Y. (2023). Love me, love my dog: does destination attractiveness not only mitigate tourists’ anger and regret emotions but also prevent negative word of mouth? *Current Issues in Tourism*, *26*(3), 1–19. <https://doi.org/10.1080/13683500.2022.2080647>
196. Yin, J., Liu, L., & Ni, Y. (2025). Melodic journeys: How place distinctiveness perception of destination songs inspires information-seeking behavior. *Journal of Hospitality and Tourism Management, 65,* 101338. https://doi.org/10.1016/j.jhtm.2025.101338
197. Yin, J., Qu, X., & Ni, Y. (2024). The marketing of destination distinctiveness: the power of tourism short videos with enjoyability and authenticity. *Current Issues in Tourism*, *27*(14), 1–13. <https://doi.org/10.1080/13683500.2023.2242559>
198. Yitao, C., Zhixi, Z., Jie, J. W., & Hongjie, S. (2024). What drives the tourist Daka? An empirical study based on the perspective of dual motivation. *Asia Pacific Journal of Tourism Research*, *29*(7), 836–851. <https://doi.org/10.1080/10941665.2024.2351109>
199. Yu, J., Dickinger, A., & Egger, R. (2024). Leveraging emojis as visual semiotics for enhanced engagement in destination marketing. *Journal of Destination Marketing & Management*, *33*(10), 100925. <https://doi.org/10.1016/j.jdmm.2024.100925>
200. Yung, R., Khoo-Lattimore, C., & Potter, L. E. (2021). Virtual reality and tourism marketing: Conceptualizing a framework on presence, emotion, and intention. *Current Issues in Tourism*, *24*(11), 1–21. <https://doi.org/10.1080/13683500.2020.1820454>
201. Yung, R., Khoo-Lattimore, C., & Potter, L. E. (2021). VR the world: Experimenting with emotion and presence for tourism marketing. *Journal of Hospitality and Tourism Management*, *46*(3), 160–171.
202. Zhang, A., & Xiao, H. (2025). Psychological well-being from virtual tourism: A mixed method approach. *Asia Pacific Journal of Tourism Research*, 1–17. <https://doi.org/10.1080/10941665.2024.2343066>
203. Zhang, H., Yang, Y., Zheng, C., & Zhang, J. (2016). Too dark to revisit? The role of past experiences and intrapersonal constraints. *Tourism Management*, *54*(2), 452–464. <https://doi.org/10.1016/j.tourman.2016.01.002>
204. Zhang, J., Zheng, C., & Qian, L. (2025). Spark aspirations: The role of mental imagery and place memories in virtual communist heritage. *Journal of Hospitality and Tourism Management, 62,* 163–172. https://doi.org/10.1016/j.jhtm.2024.12.011
205. Zhang, Q., Huang, R., Chen, Q., & Zhang, J. (2025). Balancing familiarity and novelty: the interplay of cultural familiarity and food neophilia in shaping tourists’local food experiences. *Current Issues in Tourism,* 1–23. https://doi.org/10.1080/13683500.2025.2537229
206. Zhang, H., Gordon, S., Buhalis, D., & Ding, X. (2018). Experience Value Cocreation on Destination Online Platforms. *Journal of Travel Research, 57(8)*, 1093–1107. https://doi.org/10.1177/0047287517733557
207. Zhang, W., & Fesenmaier, D. R. (2018). Assessing emotions in online stories: Comparing self-report and text-based approaches. *Information Technology & Tourism*, *20*(1-4), 83–95. <https://doi.org/10.1007/s40558-018-0122-y>
208. Zhang, X., Chen, Z., & Jin, H. (2021). The effect of tourists’ autobiographical memory on revisit intention: does nostalgia promote revisiting? *Asia Pacific Journal of Tourism Research*, *26*(9), 1–20. <https://doi.org/10.1080/10941665.2020.1718171>
209. Zhiwei (CJ) Lin, Ruan, H., Zhang, H., & Xiwen (Sivin) Xiao. (2024). Antecedents, manifestations, and environmental consequences of destination anthropomorphism: The case of nature-based destinations. *Journal of Hospitality and Tourism Management*, *58*(2), 432–444. <https://doi.org/10.1016/j.jhtm.2024.03.001>
210. Zhou, S., Yan, Q., Yan, M., & Shen, C. (2020). Tourists’ emotional changes and eWOM behavior on social media and integrated tourism websites. *International Journal of Tourism Research*, *22*(3). <https://doi.org/10.1002/jtr.2339>
211. Zhang, Y., Shi, S., Liu, N., & Leung, W. K. S. (2025). From virtual to reality: how online gamification applications promote travel intention. *Journal of Travel & Tourism Marketing, 42(6),* 832–849. https://doi.org/10.1080/10548408.2025.2511659
